# Successful validation of a larval dispersal model using genetic parentage data

**DOI:** 10.1371/journal.pbio.3000380

**Published:** 2019-07-12

**Authors:** Michael Bode, Jeffrey M. Leis, Luciano B. Mason, David H. Williamson, Hugo B. Harrison, Severine Choukroun, Geoffrey P. Jones

**Affiliations:** 1 School of Mathematical Sciences, Queensland University of Technology, Brisbane, Australia; 2 Australian Museum Research Institute, Sydney, Australia; 3 The Institute for Marine and Antarctic Studies, University of Tasmania, Hobart, Australia; 4 ARC Centre of Excellence for Coral Reef Studies, James Cook University, Townsville, Queensland, Australia; 5 College of Science and Engineering, James Cook University, Townsville, Queensland, Australia; University of California, UNITED STATES

## Abstract

Larval dispersal is a critically important yet enigmatic process in marine ecology, evolution, and conservation. Determining the distance and direction that tiny larvae travel in the open ocean continues to be a challenge. Our current understanding of larval dispersal patterns at management-relevant scales is principally and separately informed by genetic parentage data and biological-oceanographic (biophysical) models. Parentage datasets provide clear evidence of individual larval dispersal events, but their findings are spatially and temporally limited. Biophysical models offer a more complete picture of dispersal patterns at regional scales but are of uncertain accuracy. Here, we develop statistical techniques that integrate these two important sources of information on larval dispersal. We then apply these methods to an extensive genetic parentage dataset to successfully validate a high-resolution biophysical model for the economically important reef fish species *Plectropomus maculatus* in the southern Great Barrier Reef. Our results demonstrate that biophysical models can provide accurate descriptions of larval dispersal at spatial and temporal scales that are relevant to management. They also show that genetic parentage datasets provide enough statistical power to exclude poor biophysical models. Biophysical models that included species-specific larval behaviour provided markedly better fits to the parentage data than assuming passive behaviour, but incorrect behavioural assumptions led to worse predictions than ignoring behaviour altogether. Our approach capitalises on the complementary strengths of genetic parentage datasets and high-resolution biophysical models to produce an accurate picture of larval dispersal patterns at regional scales. The results provide essential empirical support for the use of accurately parameterised biophysical larval dispersal models in marine spatial planning and management.

## Abstract 2

Our understanding of marine fish larva dispersal is currently limited by sparse data and unvalidated models; combining DNA parentage matches with an oceanographic model of fish larvae on Australia’s Great Barrier Reef allows the authors to ground-truth a vital tool for sustainably managing coral reef fisheries.

## Introduction

Most marine species have a brief pelagic larval stage that is spent dispersing in open water [1,2]. Because suitable habitat is often patchy and isolated, larval dispersal is the primary process through which populations of demersal species are connected and replenished. Accurately predicting where larvae settle at the end of their dispersal stage is essential for understanding the ecology and evolution of marine populations and species [3,4] and for making decisions about their conservation and sustainable exploitation [5–8]. To this end, recent advances in biological oceanography and genetics have provided separate and revolutionary insights into larval dispersal and population connectivity in the coastal oceans [9].

Coupled biological-oceanographic models (hereafter “biophysical” models) can comprehensively describe larval dispersal patterns, simulating the movement of billions of larvae across entire seascapes [3,10,11]. Increasingly sophisticated biophysical models, with higher resolution and incorporating knowledge of larval biology and behaviour, are widely advocated as decision-support tools for coastal ecosystem management [12–16]. Although the oceanographic components of these models are usually validated, their biological components are often underdeveloped and uncertain [17,18]. The resulting model predictions are therefore of unknown accuracy and precision [19].

Genetic parentage techniques have recently been applied to directly measure larval dispersal patterns at both local and regional scales [7,20–22]. Parentage data have provided evidence for local retention of larvae [20] and for multidirectional dispersal over hundreds of kilometres [21,22]. They have also supported marine spatial management and have informed the design of marine reserve networks [7,23]. Although parentage assignments provide strong evidence of specific dispersal events, constraints on the spatial and temporal scales of population sampling mean that only a fraction of potential dispersal pathways can be resolved. Nonetheless, parentage datasets have the potential to provide an independent test of biophysical model predictions and to decide whether these models provide credible representations of true larval dispersal patterns [9].

The validation of biophysical models with parentage data is a crucial next step in marine spatial ecology and conservation [2,5,19,24–26]. Genetic parentage data provide an opportunity to validate biophysical models at the spatial and temporal scales that are most relevant to management, using the distance, direction, and timing of known dispersal events. Given that both biophysical models and parentage data describe the sources and destinations of individual larvae, the process of statistical validation is conceptually straightforward. However, it is logistically demanding to assemble the necessary data for several reasons. First, genetic parentage assignments are expensive to obtain at the requisite intensity and spatial scale [9]. If there are too few assignments, the parentage dataset may not offer the statistical power needed for a rigorous test [27,28]. Second, it is challenging to produce a biophysical model at both the resolution and scale needed to match observed assignments, as the observed dispersal events include both small-scale retention of larvae in and around their natal population and long-distance connections over hundreds of kilometres. Third, because ocean currents are often highly variable through time, the model must provide a close temporal match to the measured larval dispersal events [19]. Finally, to isolate the significance of larval biology and behaviour, parentage data should be tested against a range of different biophysical models, from passive dispersal models to those incorporating the biological and behavioural traits of the study species [17,18,25].

Here, we resolve each of these challenges to validate a biophysical model with a matching genetic parentage dataset. We parameterise a high-resolution biophysical model of larval dispersal across the southern and central Great Barrier Reef (GBR) for bar-cheeked coral trout (also called the spotted coral grouper, *P*. *maculatus*, Serranidae). We develop novel quantitative validation techniques and use them to rigorously test this model against an intensive and independent genetic parentage survey of *P*. *maculatus* (previously published in [21]). Our results represent a formal validation of a regional-scale biophysical model using genetic parentage data, thereby integrating two important and complementary sources of larval dispersal information.

## Materials and methods

### Genetic parentage assignments and population sampling

**Fig 1:**
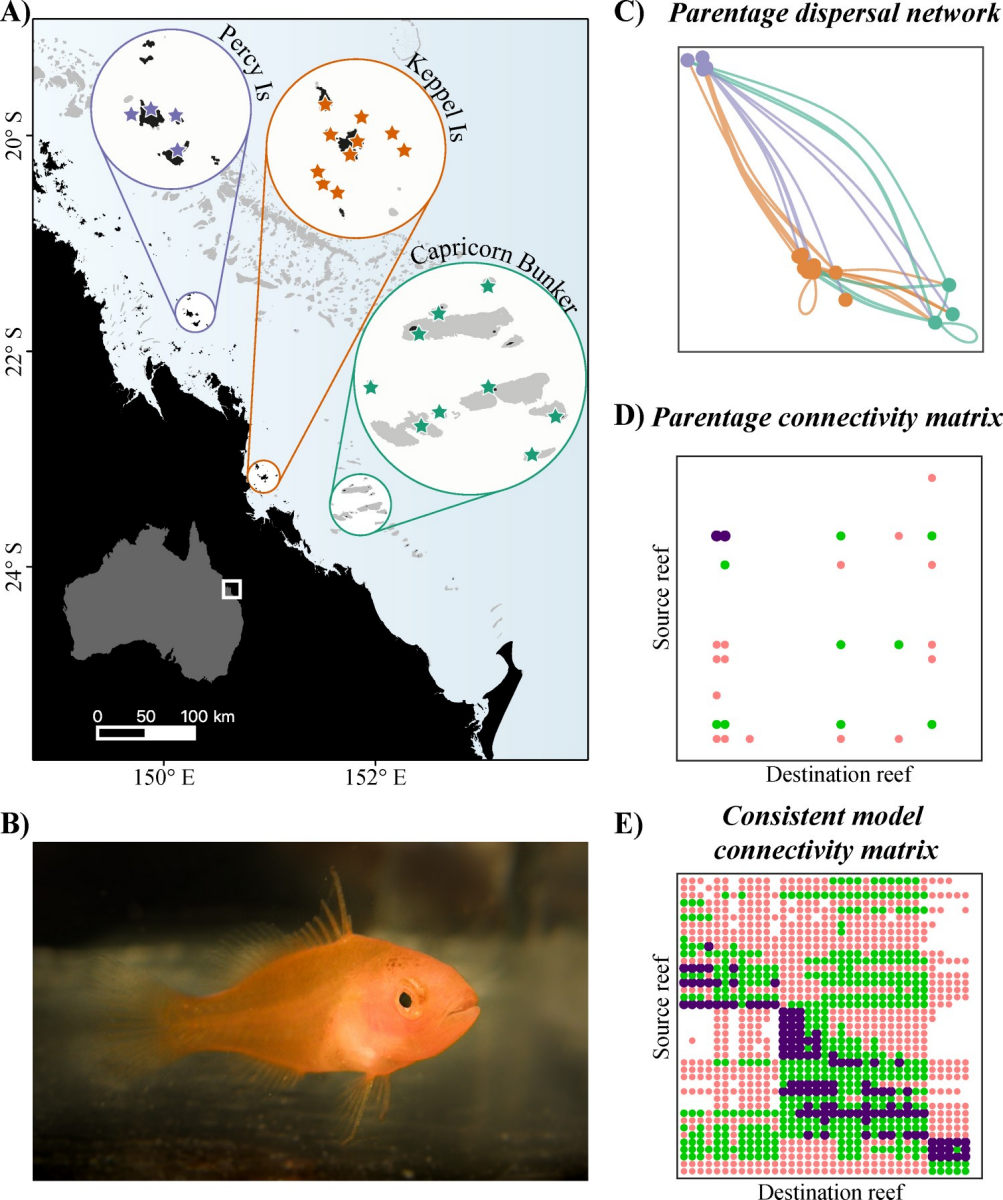
Study region and data. (A) Location of the study region in the southern Great Barrier Reef, Australia. Stars highlight sampled reefs in each region. (B) 16-mm *P*. *maculatus* settlement-stage larva. *Photo credit*: *C*. *Wen*. (C) Observed connectivity network between the sampled reefs. Line and reef colours indicate the identity of the source region. Note that bidirectional connections were observed between all three regions. (D) Parentage connectivity matrix for the sampled reefs. Only a few rows and columns contain connections, because only a subset of reefs were sampled. (E) Connectivity matrix generated by the consistent biophysical model simulations, for all reefs in the three regions. Each row and column correspond to a reef in one of the three sampled regions. In both matrices, colours indicate the relative strength of dispersal, with purple > green > red. Biophysical simulations give a more complete picture of regional dispersal patterns, but the genetic parentage data are empirically defensible. The data used in this figure are given in S1 Data. Is, Islands.

Between September 2011 and August 2013, a total of 880 adult and 1,190 juvenile *P*. *maculatus* were sampled across three reef regions in the southern GBR: the Keppel Islands, the Percy Islands, and the Capricorn Bunker group (Fig 1A and 1B). A panel of 23 microsatellite loci identified 69 parent–offspring relationships within the sample, connecting 18 different patch reefs separated by distances ranging from a few hundred metres to over 200 km. The observed larval dispersal network revealed self-recruitment within each region (including to individual natal reefs) and longer-distance multidirectional dispersal between regions (Fig 1C), creating an observed parentage dispersal matrix (Fig 1D). Each assigned juvenile and a subset of 314 unassigned juveniles were aged from daily otolith growth rings to determine both the spawn date and the pelagic larval duration. This allowed a closer parameterisation of the biophysical model and precise temporal match between empirical dispersal vectors and the biophysical model.

Underwater visual census (UVC) surveys were conducted on reefs within each of the three regions to establish reef-specific estimates of adult population size. This was combined with *P*. *maculatus* fecundity data to estimate the number of propagules released from each reef in the biophysical model. The species’ relative abundance was highly variable among reefs, particularly between reserve and nonreserve reefs. For reefs within the study domain where populations were not surveyed, adult densities were approximated by the average density of surveyed reefs with the same protected status within their region. The biophysical model used these population estimates to account for the unassigned juveniles during model fitting. For full details of the population sampling and genetic parentage assignments, see S1 Text and reference [21].

### Biophysical larval dispersal model

The biophysical model was physically and biologically parameterised for the locations and timing of the genetic parentage study. The southern GBR has one of the most complex bathymetries on the east Australian coast. A dramatic narrowing of the continental shelf marks the end of the GBR, leaving its reef systems particularly exposed to oceanic influences. The Capricorn Channel divides two geomorphologically distinct coral reef systems—the Percy Islands, Keppel Islands, and Capricorn Bunker group in the inshore and midshelf and the dense Swain Reefs on the outer shelf (Fig 1A and S2 Text).

Surface currents are predominantly driven northwest by strong trade winds. However, flows become more variable during the austral summer when *P*. *maculatus* spawning is most intense (S2 Text), when tropical lows can cause southward reversals [29,30]. Circulation is also driven by the poleward-flowing East Australian Current, a western boundary current that flows strongest during the summer. The East Australian Current periodically generates strong cyclonic eddy structures, which create mesoscale recirculation patterns that have a particular influence on flow in the Capricorn Channel [31].

These flows are captured by the hydrodynamic component of the biophysical model. The model is based on a temporally implicit 3D barotropic scheme, built from three nested computational grids with resolutions of 1.85 km (1 nautical mile) for the whole GBR, 370 m for the three sample regions, and 74 m around focal reefs. The numerical scheme was developed from the models of James and colleagues [10] and Luick and colleagues [32]. It incorporates a subgrid scale parameterisation of hydrodynamic impedance around GBR reefs that results in more accurate modelling of currents passing through the complex matrix of reefs in the GBR lagoon. Currents were determined hourly throughout the period July 2011 to July 2013, which encompasses all dispersal events in the parentage dataset.

The biological larval component is an individual-based model that tracks each propagule in 5-minute time steps from spawning to settlement or mortality. Its behavioural assumptions incorporate the recommendations of North and colleagaues [33] and Staaterman and Paris [17] and include the buoyancy of pelagic eggs, realistic larval sensory ability, behaviour (ontogenetic vertical migration, swimming performance, and orientation), pelagic larval duration and mortality, and adult spawning phenology. Importantly, all of these behaviours exhibit diel, spatial, and ontogenetic variation. They also exhibit individual variation, with the behaviour of each individual larva being sampled from probability distributions. Behavioural parameters were based on empirical data for *P*. *maculatus* or the most closely related grouper species for which the required information is known. For each new moon period between July 2011 and July 2013, we simulated the release of eggs from all reef slope habitats within the study domain, creating 24 biophysical dispersal matrices (Fig 1E). To achieve the best estimates of the probabilities contained in these matrices, we released as many eggs at each spawning event as computationally possible (more than 25 million per event).

We repeated these simulations using three different behavioural variants of the model. Two models were plausible representations of *P*. *maculatus* larval behaviour, based on best estimates for each parameter: a “consistent” behaviour model, in which each larva’s behaviour was consistent within each ontogenetic stage, based on a single sample from each parameter’s probability distribution, and a “varying” behaviour model, in which the behaviour of each larva varied within and between ontogenetic stages, by resampling from each distribution. For example, the average larvae in the consistent and varying models swim at the same depth. However, in the consistent model, a particular larva would maintain a single swimming depth during each ontogenetic stage aside from diel vertical movements, whereas in the varying model, that larva would sample a range of depths over time. Both models are a reasonable interpretation of observational evidence. The final behavioural variant was a “passive” behaviour model, in which larvae act as neutrally buoyant particles with minimal behaviour (larvae in the consistent and varying models control vertical distribution from hatching and develop horizontal swimming abilities once the caudal fin forms). Our primary goal in simulating the three different behavioural models was not to draw conclusions about specific behavioural parameters, since this would require a much larger set. Rather, we wanted to assess the importance of including larval behaviour (i.e., the passive model, compared with the consistent and varying models) and to determine whether the parentage dataset had enough statistical power to discriminate between different plausible behavioural assumptions (i.e., the varying model, compared with the consistent model). For full details of the hydrodynamic and biological components of each of the biophysical larval dispersal models, see S2 Text.

## Results

### Matching biophysical models with empirical parentage data

Biophysical model predictions can be compared with parentage data in different ways, and the amount of agreement will vary between goodness of fit tests. To ensure a robust validation, we performed multiple comparisons between the observed parentage data and the biophysical simulations, with a focus on the scale and direction of larval dispersal—factors that have been identified as important for marine science and management. For full details of each goodness of fit test, see S3 Text.

#### Matching individual larval dispersal events

An obvious initial goodness of fit test is whether the biophysical models can reproduce the particular dispersal events observed in the parentage dataset, including bidirectional dispersal between reef regions (Fig 1C). To give one example, the parentage dataset contains a larva that was sampled on South Island in the Percy Islands, whose parents lived on Polmaise Reef in the Capricorn Bunker group. This larva therefore dispersed more than 230 km (straight-line distance) from its natal reef, and otolith analysis indicates that this dispersal occurred at the end of November 2011. When we look for this same event in the consistent biophysical model simulations, we find that it occurred multiple times (S3 Text).

We repeated this process for each of the 69 assigned juveniles in the parentage dataset, searching the three biophysical models for matching events. The consistent biophysical model delivered the most matches. Altogether, 66 of the observed dispersal events in the parentage dataset could be spatiotemporally matched to simulated events in this model. The remaining three dispersal events were observed in the consistent model simulations, but not on the precise spawning dates indicated by the otolith analysis (i.e., they were spatially but not temporally matched). This degree of event matching is highly unlikely by random chance (S3 Text). Furthermore, neither the varying nor the passive model achieved this level of matching; in fact, neither could recreate the majority of observed events (32 and 34 events could be matched, respectively).

#### Maximum-likelihood model validation

**Fig 2:**
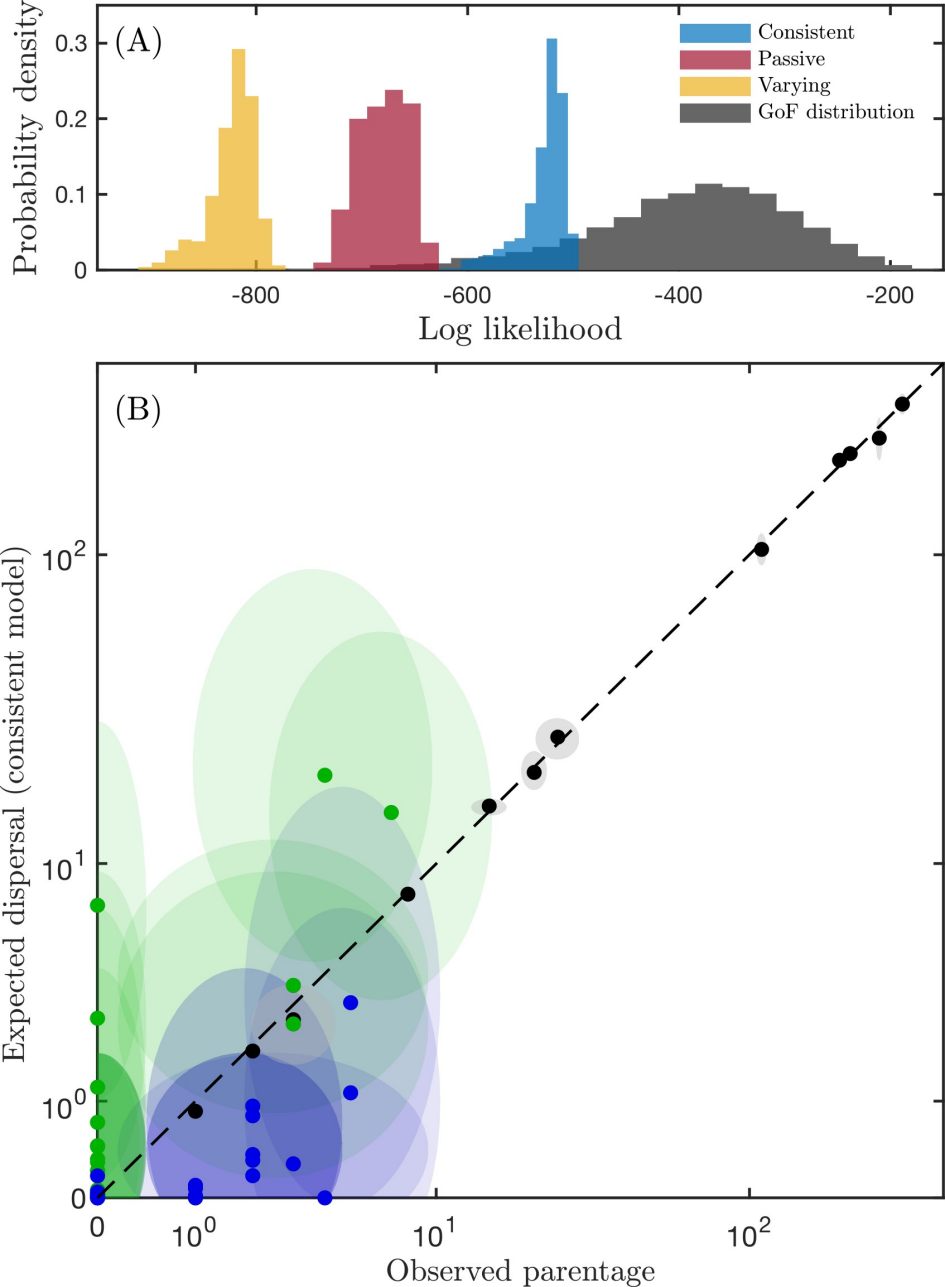
Biophysical model fit. (A) Histograms show probability distributions of the likelihood of the three biophysical models, given the dataset of genetic parentage assignments. The consistent model (blue) provides the unambiguous best fit to the data. The spread of each distribution is caused by sampling and uncertainty about adult population sizes. The overlap of the consistent model with the parametric GoF distribution (shown in grey) indicates a good fit; the lack of overlap for the varying and passive model show them to be poor fits. (B) Scatterplot of observed parentage assignments against the expected number of assignments according to the consistent model. Dashed line shows the 1:1 fit line. Shaded ellipses indicate uncertainty, estimated using multinomial confidence bounds for the parentage data, and random subsampling for the biophysical data. Green indicates assignments within the same group of reefs; blue indicates assignments between reef groups, and black indicates unassigned juveniles (i.e., juveniles whose parents were not among the sampled adults). The data used in this figure are given in S1 Data. GoF, goodness of fit.

To assess which model provided the best fit to the empirical data, we calculated the likelihood that the observed parentage assignments were generated by each of the three candidate biophysical models. A maximum-likelihood framework can be applied by assuming that biophysical model simulations approximate the multinomial probability distributions of larval destinations from each source reef. Our likelihood function (S3 Text) incorporates the sampling intensity for both juveniles and adults, the distribution of unsampled reefs in the metapopulation, variation in adult populations between source reefs, postsettlement mortality, and the proportion of sampled juveniles that could not be assigned to any genotyped adults, all of which influence model fit [26]. The results showed that the consistent model produced the best fit to the observed data and convincingly outperformed both the passive and the varying models (Fig 2A).

The consistent model provides reasonable predictions of the relative strength of dispersal between reefs in the southern GBR and accurate predictions of the unassigned juveniles sampled on each reef (Fig 2B). The confidence bounds around each observation reflect the sampling variance inherent in parentage datasets, in which assigned juveniles represent a small proportion of the total recruitment to a population. They also include the uncertainty around our estimates of the adult population size of (and thus the larval output from) each reef. The consistent biophysical model tends to overestimate the amount of dispersal within the regions (green markers), which includes self-recruitment, and underestimate the amount of dispersal between the regions (blue markers). It predicts a number of within-region short-distance dispersal events that were not observed (green markers along the y-axis). This is unexpected, as juvenile sampling is often biased towards locations near sampled adult populations [9,20], making short-distance dispersal events easier to observe. The two other biophysical models provided poor fits to the data; the varying model predicted almost no between-region recruitment, whereas the passive model underestimated both within- and between-region recruitment (S3 Text).

As well as producing the best fit to the data, a parametric bootstrap goodness of fit test indicated that the consistent model produced a statistically good fit to the parentage data. Fig 2A shows that the observed likelihood values fall within the 95% confidence bounds of expected likelihoods, if the biophysical model represents the true dispersal process (S3 Text). In other words, the consistent model would produce parentage data that are indistinguishable from our empirical dataset; this suggests that the consistent model provides an accurate representation of the species’ larval dispersal in the southern GBR. According to this same goodness of fit test, neither the varying nor the passive model provided good fits to the parentage data (Fig 2A).

We ran a post hoc power analysis to determine whether the likelihood method in general, and our parentage assignment dataset in particular, were powerful enough to discriminate between alternative biophysical models. When faced with a simulated parentage assignment dataset that was sampled from one of our three biophysical models (at the same intensity as the observed dataset), we were able to identify the correct model 83% of the time (see S3 Text for details). The consistent and passive models were correctly identified from 97% and 94% of parentage datasets, but the varying model was harder to identify. It was correctly identified from 58% of parentage datasets, whereas the remainder were misclassified as the consistent model.

Following previous analyses [34,35], we also calculated the correlation between the observed parentage matrix and equivalent matrices sampled from the three biophysical simulation models. We found a consistently high correlation between the observed data and all three biophysical models (Pearson’s *r* > 0.95), suggesting that this type of test is not useful for identifying the best model.

#### Larval dispersal scale and direction

The distance and direction of larval dispersal are important elements in ecological and evolutionary theory and are central parameters in marine spatial management. They drive source-sink dynamics [36], economic externalities [6,37], and the design and performance of marine reserve networks [7,38]. Useful biophysical models must provide an accurate estimate of these metrics.

**Fig 3:**
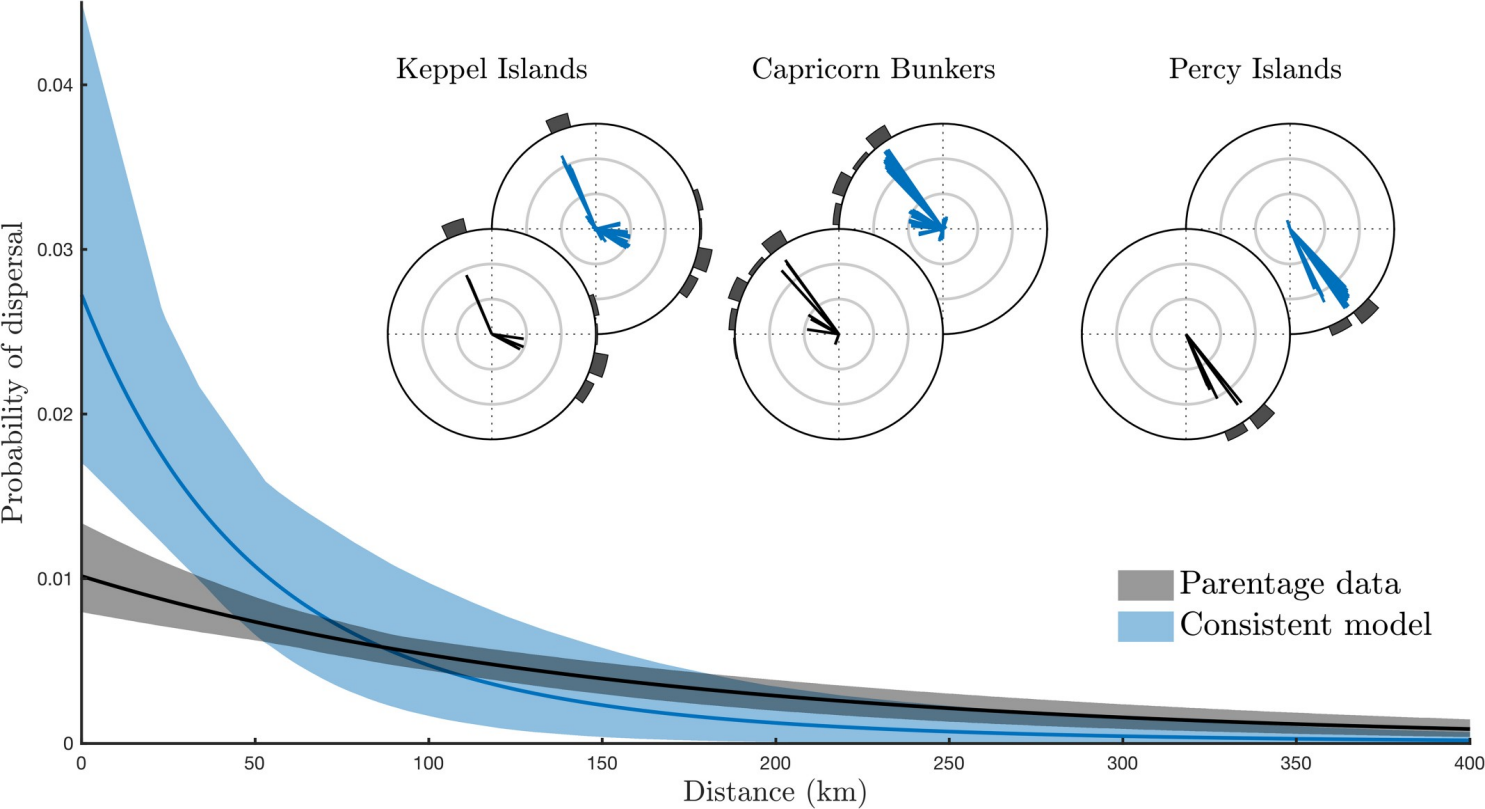
Scale and direction of dispersal. Dispersal kernels indicating the scale of dispersal observed in the dataset of parentage assignments (grey) and the consistent model simulations (blue). Shaded regions indicate 95% confidence bounds of uncertainty, resulting from sampling variation (adults and juveniles) and uncertain adult population size. The consistent model predicts a larger proportion of short-distance dispersal—and consequently, less long-distance dispersal—than the genetic parentage data. Inset: compass plots illustrating the straight-line direction and distance travelled from each reef group by all larvae in the parentage dataset (black) and in the consistent model (blue). Concentric circles measure distances of 100 km, 200 km, and 300 km (outside circle). Histograms indicate the relative availability of sampled habitat in each direction in the other two regions. The data used in this figure are given in S1 Data.

To measure the distance scale of recruitment predicted by the consistent biophysical model and observed in the parentage assignments, we fit isotropic larval dispersal kernels to both [26]. The two kernels broadly agree, although the consistent model simulations were best fit by a shorter dispersal kernel than the observed parentage data, and this difference was particularly apparent at distances of less than 50 km (Fig 3). However, both kernels readily predicted both within-region connections and also the 200+ km connections observed between different regions.

Fig 3 also shows that the consistent biophysical model recreates the directional elements of the parentage dataset for each of the three sampling regions. Neither the passive nor the varying models were able to achieve this (S3 Text). This result also illustrates the influential role played by larval behaviour in determining dispersal patterns: although all three models were forced by the same underlying oceanography, the parameterised larval behaviour of the consistent model was needed to accurately predict the directional patterns of observed larval dispersal.

## Discussion

Our results compare three variants of a high-resolution biophysical larval dispersal model (consistent, passive, and varying) with high-quality genetic parentage data, using formal statistical comparisons. A suite of tests indicate that our sample of 1,190 *P*. *maculatus* juveniles—including 69 positive parentage assignments—could plausibly have been generated by the consistent biophysical model. This match holds across several different types of comparison, including event matching, model likelihood, and aggregate estimates of dispersal distance and direction. The goodness of fit is strengthened by the fact that the parentage dataset was powerful enough to statistically exclude two alternative variants of the biophysical model. The poor fit of the passive model confirms the importance of including larval behaviour and ontogeny in biophysical models, whereas the poor fit of the varying model emphasises the importance of including the correct larval behaviour.

Our analysis only considered the fit of three biophysical models from among the very large number that could be created using different values of pelagic larval duration, larval growth rates, swimming ability, and so on. It is possible that one of these untested biophysical models offers a better fit than the consistent model. However, it is important to note that the consistent model is mechanistic and deductive, constructed using direct empirical measurements of model parameters. As a consequence, the superior fit of a model with different (and therefore less realistic) values would not necessarily mean that it offers a better description of *P*. *maculatus* larval dispersal. When confronted with independent data on larval dispersal that was gathered using a completely different technique (genetic parentage), the consistent biophysical model provided a reasonable fit along a number of important dimensions. Despite some important deviation, particularly across short dispersal distances (Fig 3), these results represent a validation of this biophysical model.

Quantitative comparisons between biophysical simulations and known dispersal trajectories for marine fish are challenging, requiring accurate biophysical predictions over distances that range across several orders of magnitude (10^2^–10^5^ m in our parentage dataset). Past attempts to compare biophysical simulations with parentage data have been limited by small numbers of assignments and informal, qualitative comparisons [27,28,39]. None attempted to compare alternative biophysical models, and without the use of formal statistical methods, inadequate models cannot be rejected, goodness of fit cannot be assessed, and we cannot be confident that any apparent match is not a statistical artefact.

A number of other analyses have compared biophysical models of larval dispersal with different kinds of empirical data, although very few did so quantitatively [34]. Most have been based on recreating genetic distance matrices [11,34,40], but this type of population genetic structure is only evident at large spatial scales and across many generations of dispersal. This makes genetic distance spatially and temporally poorly suited for biophysical model validation [2]. Other comparisons used assignments tests, which identify dispersal trajectories by matching larval signatures (genetic or geochemical) to source patches [34,35,41]. Regional assignment matrices can be closely correlated with biophysical matrices, but it can be difficult to positively assign larvae at smaller scales (site-level misclassification rates can exceed 50%), because genetic and geochemical differentiation is generally limited between patches in a marine metapopulation [34,42]. This limits the strength of the validation they offer biophysical models for marine spatial management purposes, in which the most important decisions must be made at precise scales [19].

Our analyses integrate biophysical simulation models with genetic parentage data—two descriptions of larval dispersal that have come to dominate marine dispersal research. Each data type has unique strengths: biophysical models are cost-effective and can offer a complete picture of larval dispersal in a region, whereas parentage analyses offer the best direct evidence dispersal at management-relevant scales. When the spatiotemporal coverage of biophysical model predictions is reinforced by the evidence contained in genetic parentage dataset, the result is strongly complementary, opening new avenues in larval dispersal research. For example, metapopulation viability models require estimates of dispersal between reefs, and particularly local retention—the amount of reproductive output that returns to the natal population [5,8,24]. All presently available empirical data, including parentage assignments, can only estimate the amount of self-recruitment [24], but empirically validated biophysical models can effectively translate observations of recruitment into conclusions about dispersal. Parentage analyses and biophysical modelling can also inform each other by using larval dispersal simulations to guide and prioritise empirical sampling and by using parentage data to understand where and why model predictions fail. Finally, validated biophysical models can help explain unusual events in parentage assignment datasets. For example, the GBR dataset contains simultaneous long-distance dispersal in different directions—from the Percy Islands to the Capricorn Bunker group, for instance. These events are common in the consistent biophysical model simulations, and inspection reveals that the larvae are likely moving under the influence of contrasting inshore and offshore currents (S3 Text; S1 Animation).

The observed match between an extensive genetic parentage dataset and a high-resolution dispersal simulation represents an important and overdue validation of biophysical models. Biophysical models are the foundation of important new theories in marine ecology and evolution, and their predictions are being incorporated into decision-support tools for marine spatial planning and policy. Given this widespread usage, establishing the credibility of these models is a matter of urgency. Our observed match between models and data in the southern GBR provides reassuring support for existing applications of biophysical models worldwide, but also directions for future improvement. The methods we have developed provide a template for such future validation efforts.

## Supporting information

S1 Text Full description of the genetic parentage dataset, including the adult abundance surveys. (DOCX)

S2 Text Full description of the biophysical larval dispersal model, including the oceanographic and biological components. (DOCX)

S3 Text Full description of the statistical matching methods, including event matching, likelihood model, directional matching, and dispersal kernel fitting. (DOCX)

S1 Animation Animation of long-distance bidirectional larval dispersal in the Southern Great Barrier Reef. Simulations are generated by the consistent biophysical model, recreating the 26/07/2011 spawning event. Red larvae were spawned in the Capricorn Bunker group; blue larvae were spawned in the Percy Islands. Note that these larvae were selected for illustrative purposes and are neither all the larvae that were released from these two reef groups nor the only ones that travelled between them.
(GIF)

S1 Data Spreadsheet containing the data illustrated in Fig 1, Fig 2 and Fig 3. (XLSX)
