# Supplementary material for: Successful validation of a larval dispersal model using genetic parentage data
Source: PLoS Biol. 2019 Jul 12;17(7):e3000380. doi: 10.1371/journal.pbio.3000380 (PMC6655847; doi:10.1371/journal.pbio.3000380)
Supplement: S1 Text — (DOCX) [file pbio.3000380.s001.docx]

**Supporting Information Text 1: Empirical data**

**Study locations**

Empirical data was collected from the Keppel Islands (23° 10’ S, 150° 57’ E), the Percy Islands (21° 42’ S, 150° 18’ E), and the Capricorn bunker group (23° 25’ S, 151° 46’ E) in the southern section of the Great Barrier Reef (GBR), Australia (Figure 1A, main text). The Keppel and Percy Island groups are archipelagos of high continental islands surrounded by fringing coral reefs, while the Capricorn Bunker group comprises emergent platform reefs located on the outer margin of the continental shelf. The vast majority of the seafloor surrounding the three focal reef clusters is dominated by open sand habitat; apart from several deep-water shoals to the north of the Capricorn Bunkers, there are no other significant coral reef habitats within the study domain.

At the Keppel Islands, fringing coral reefs cover approximately 700 ha, of which 196 ha (~ 28%) is protected within a network of no-take marine reserves. The Percy Islands are surrounded by approximately 1870 ha of fringing coral reefs. The Capricorn Bunker group is comprised of a vast area of platform reefs, many of which have extensive reef flat and lagoon habitats. This study focused on eleven reefs in the northern section of the Capricorn Bunkers, bound by Polmaise Reef (southwest), Northwest Reef (northwest), North Reef (northeast), and One Tree Reef (southeast). The eleven focal reefs have a total reef area of nearly 25,700 ha, of which approximately 14,700 ha (~ 57 %) is designated as no-take marine reserve and 11,000 ha (~ 43 %) is open to fishing.

**Study species**

Bar-cheek coral trout (or the spotted coral grouper, *Plectropomus maculatus,* Serranidae) were the focal species of this study. *P. maculatus* inhabit coral reefs throughout the central Indo-Pacific region. Across this distribution, and particularly within the GBR, *P. maculatus* are generally most abundant on inner-shelf and fringing reefs, but are rare on outer-shelf reefs (1), where their ecological niche is filled by their congenerics, *P. leopardus* and *P. laevis*. The specific distribution and abundance patterns of *P. maculatus* are important when estimating larval contributions from unsampled reefs.

All three coral trout species are heavily exploited throughout their geographic ranges; in the GBR Marine Park, *P. maculatus* are targeted by both recreational and commercial fishers (2). Rapid and sustained increases in mean density, body size and biomass of coral trout have been recorded on reserve reefs throughout the GBR Marine Park, while populations on non-reserve reefs have remained relatively stable (3, 4)**.** The protection status of unsampled reefs is thus an important factor to consider when estimating their contribution to larval recruitment.

Like many other grouper species, *P. maculatus* can form large spawning aggregations at predictable locations and times in some regions, while small group spawning has also been documented (5, 6). The species is polygynous, and large males actively exclude subordinate males from spawning sites (5). The peak spawning period for coral trout in the GBR is during the Austral summer (September – February). This distribution of spawning times can be seen in our dataset (*Supporting Information Text S2;* Figure S2.5A). Most spawning events occur around dusk on several days either side of the new moon lunar phase (7). Aside from relatively short (generally less than 10 km) migrations to aggregation sites, adult coral trout occupy distinct home ranges in the order of 1–4 ha and rarely move among reefs separated by expanses of open sand or low-relief benthic habitat (8 – 10) .

**Estimation of coral trout density, population size and the effects of reserves**

Underwater visual censuses (UVC) were conducted on reefs throughout the three focal regions to quantify *P. maculatus* densities and to provide a baseline for estimating total population size. A towed-GPS UVC method was used, with replicated 10-minute UVC tracks surveyed within reef slope, reef crest, reef flat and lagoon habitats on each focal reef. Observers recorded the number and estimated the total length (5 cm categories) of all *P. maculatus* sighted on each UVC track. Mean densities of adult *P. maculatus* were calculated for each reef within the study domain (see 11, *supplementary material*).

**Sampling of coral trout populations**

A total of 880 adult and 1,190 juvenile *P. maculatus* were sampled from reefs in the three regions between September 2011 and August 2013. Both reserved reefs (“no-take” Marine National Park zones) and fished reefs (Conservation Park Zones and Habitat Protection Zones) were sampled in each of the three regions. Approximately 61% of the adult samples and 38% of the juvenile samples were collected from reserve reefs. All remaining samples were collected from non-reserve reefs. The proportion of adult *P. maculatus* populations sampled on focal reefs ranged from approximately 1% to 21% (11).

**Genetic parentage analyses**

Adult and juvenile *P. maculatus* were genotyped for parentage analysis using a panel of 25 microsatellite loci previously described for this system (12). Estimates of locus heterozygosity, Hardy-Weinberg expectations and the probability of excluding false parent-offspring pairs were measured for each locus on adult samples only to avoid the effect of related individuals in the data. Two loci were removed due to departure from Hardy-Weinberg expectation or the presence of rare alleles. The average number of alleles of the remaining 23 loci was 18.7, with an average observed heterozygosity of 0.796, and a cumulative exclusion probability of 1 – 2.31 x 10^-7^ (Table S1.1).

*Table S1.1. Diversity indices of 23 microsatellite loci used in the assignment of parent-offspring pairs of bar-cheek coral trout (P. maculatus) in the southern Great Barrier Reef. The number of observed alleles (Na), observed (Ho) and expected (He), fixation index (F), the exact test for Hardy-Weinberg expectation (HWE) and probability of exclusion (PE) were estimated from 880 adult genotypes.*

| **Locus** | **Na** | **Ho** | **He** | **F** | **HWE** | **PE** | **1-CumPE** |
| --- | --- | --- | --- | --- | --- | --- | --- |
| Pma043 | 23 | 0.787 | 0.813 | 0.032 | 0.996 | 0.487 | 5.13E-01 |
| Pma097 | 29 | 0.886 | 0.890 | 0.005 | 0.995 | 0.644 | 1.82E-01 |
| Pma104 | 42 | 0.928 | 0.925 | -0.004 | 0.001 | 0.737 | 4.80E-02 |
| Pma106 | 21 | 0.854 | 0.864 | 0.012 | 0.001 | 0.578 | 2.02E-02 |
| Pma109 | 18 | 0.845 | 0.833 | -0.015 | 0.027 | 0.524 | 9.63E-03 |
| Pma114 | 16 | 0.801 | 0.822 | 0.026 | 0.010 | 0.505 | 4.77E-03 |
| Pma180 | 22 | 0.785 | 0.791 | 0.008 | 0.105 | 0.467 | 2.54E-03 |
| Ple02 | 16 | 0.857 | 0.859 | 0.003 | 0.848 | 0.563 | 1.11E-03 |
| Ple04 | 12 | 0.752 | 0.741 | -0.015 | 0.201 | 0.348 | 7.24E-04 |
| Pma012 | 12 | 0.697 | 0.720 | 0.032 | 0.018 | 0.323 | 4.90E-04 |
| Pma025 | 23 | 0.910 | 0.909 | -0.001 | 0.570 | 0.688 | 1.53E-04 |
| Pma038 | 22 | 0.881 | 0.885 | 0.004 | 0.024 | 0.634 | 5.59E-05 |
| Pma090 | 11 | 0.734 | 0.734 | 0.000 | 0.478 | 0.351 | 3.63E-05 |
| Pma101 | 16 | 0.677 | 0.671 | -0.009 | 0.998 | 0.291 | 2.57E-05 |
| Pma412 | 10 | 0.635 | 0.638 | 0.004 | 0.001 | 0.242 | 1.95E-05 |
| Ple01 | 24 | 0.749 | 0.755 | 0.008 | 0.001 | 0.401 | 1.17E-05 |
| Ple05 | 9 | 0.661 | 0.669 | 0.012 | 0.979 | 0.248 | 8.78E-06 |
| Pma020 | 22 | 0.887 | 0.885 | -0.002 | 0.023 | 0.635 | 3.20E-06 |
| Pma022 | 9 | 0.683 | 0.701 | 0.025 | 0.045 | 0.309 | 2.21E-06 |
| Pma027 | 30 | 0.844 | 0.855 | 0.013 | 0.986 | 0.562 | 9.69E-07 |
| Pma121 | 17 | 0.763 | 0.767 | 0.005 | 1.000 | 0.389 | 5.92E-07 |
| Pma191 | 11 | 0.863 | 0.849 | -0.017 | 0.740 | 0.535 | 2.75E-07 |
| Pma228 | 14 | 0.522 | 0.532 | 0.019 | 1.000 | 0.161 | 2.31E-07 |
|  |  |  |  |  |  |  |  |

All collected juveniles were screened against the total pool of adult samples to reveal parent-offspring relationships, which were identified using a maximum likelihood approach implemented in the software program famoz (13, 14). The program computes log of the odds ratio (LOD) scores for assigning individuals to candidate parents based on the observed allelic frequencies at each locus. Minimum LOD score thresholds for accepting assignments to single parents and parent pairs were determined from the distribution of Monte Carlo simulated LOD scores from 50,000 known parent-offspring pairs and 50,000 unrelated pairs. Parentage test simulations estimated the probability of falsely accepting (false positive – type I error) or excluding (false negative – type II error) parent–offspring pairs associated with these LOD thresholds (11). The resulting probability of assigning a juvenile to a parent that was not its true parent, knowing that the true parent was not sampled given a LOD threshold of 4, was 0.63% (type I error). Conversely, the probability of a true parent–offspring pair not being identified knowing that the true parent was sampled was <0.01% (type II error). We identified 69 parent-offspring pairs in the data with LOD scores ranging from 4.04 to 25.55 and an average LOD score across all assignments of 6.78 ± 0.38 S.E. (Table S2.2). All positive assignments were to single parents only (11).

*Table S2.2. Source and destination of parent-offspring pairs identified in a sample of 880 adult and 1,190 juvenile bar-cheek coral trout (P. maculatus) in the southern Great Barrier Reef and LOD scores associated with each positive assignment.*

| ***SOURCE*** |  | ***DESTINATION*** |  |  |
| --- | --- | --- | --- | --- |
| **Region** | **Reef** | **Region** | **Reef** | **LOD** |
| Capricorn Bunkers | Northwest Is | Capricorn Bunkers | Polmaise | 5.87 |
| Capricorn Bunkers | Northwest Is | Capricorn Bunkers | Polmaise | 7.58 |
| Capricorn Bunkers | Northwest Is | Capricorn Bunkers | Polmaise | 4.94 |
| Capricorn Bunkers | Polmaise | Capricorn Bunkers | Polmaise | 4.58 |
| Capricorn Bunkers | Polmaise | Capricorn Bunkers | Polmaise | 6.51 |
| Capricorn Bunkers | Polmaise | Capricorn Bunkers | Polmaise | 6.78 |
| Capricorn Bunkers | Polmaise | Capricorn Bunkers | Polmaise | 6.8 |
| Capricorn Bunkers | Northwest Is | Keppel Islands | Halfway | 5.51 |
| Capricorn Bunkers | Northwest Is | Keppel Islands | Middle Is | 7.5 |
| Capricorn Bunkers | Polmaise | Keppel Islands | Halfway | 4.85 |
| Capricorn Bunkers | Polmaise | Keppel Islands | Halfway | 5.81 |
| Capricorn Bunkers | Polmaise | Keppel Islands | Halfway | 6.01 |
| Capricorn Bunkers | Polmaise | Keppel Islands | Halfway | 9.48 |
| Capricorn Bunkers | Polmaise | Keppel Islands | Middle Is | 7.55 |
| Capricorn Bunkers | Polmaise | Keppel Islands | North Keppel | 5.48 |
| Capricorn Bunkers | Polmaise | Keppel Islands | Pumpkin Is | 4.57 |
| Capricorn Bunkers | Northwest Is | Percy Islands | Middle Percy | 5.05 |
| Capricorn Bunkers | Polmaise | Percy Islands | Middle Percy | 5.36 |
| Capricorn Bunkers | Polmaise | Percy Islands | South Percy | 5.97 |
| Capricorn Bunkers | Polmaise | Percy Islands | South Percy | 6.05 |
| Capricorn Bunkers | Polmaise | Percy Islands | South Percy | 10.47 |
| Keppel Islands | Clam Bay | Capricorn Bunkers | Erskine | 4.53 |
| Keppel Islands | Egg Rock | Capricorn Bunkers | Erskine | 6.14 |
| Keppel Islands | Egg Rock | Capricorn Bunkers | Polmaise | 10.31 |
| Keppel Islands | Halfway | Capricorn Bunkers | Northwest Is | 7.65 |
| Keppel Islands | Halfway | Capricorn Bunkers | Polmaise | 4.04 |
| Keppel Islands | Halfway | Capricorn Bunkers | Polmaise | 6.65 |
| Keppel Islands | Passage Rocks | Capricorn Bunkers | Erskine | 6.99 |
| Keppel Islands | Big Peninsula | Keppel Islands | Middle Is | 5.54 |
| Keppel Islands | Clam Bay | Keppel Islands | Clam Bay | 6.37 |
| Keppel Islands | Clam Bay | Keppel Islands | Halfway | 8.13 |
| Keppel Islands | Clam Bay | Keppel Islands | Halfway | 25.55 |
| Keppel Islands | Clam Bay | Keppel Islands | Middle Is | 4.62 |
| Keppel Islands | Clam Bay | Keppel Islands | Middle Is | 7.01 |
| Keppel Islands | Egg Rock | Keppel Islands | Halfway | 4.09 |
| Keppel Islands | Egg Rock | Keppel Islands | Halfway | 4.27 |
| Keppel Islands | Egg Rock | Keppel Islands | Halfway | 7.18 |
| Keppel Islands | Egg Rock | Keppel Islands | Middle Is | 4.51 |
| Keppel Islands | Egg Rock | Keppel Islands | Monkey Bay | 4.33 |
| Keppel Islands | Egg Rock | Keppel Islands | North Keppel | 4.27 |
| Keppel Islands | Egg Rock | Keppel Islands | North Keppel West | 13.5 |
| Keppel Islands | Egg Rock | Keppel Islands | Pumpkin Is | 8 |
| Keppel Islands | Egg Rock | Keppel Islands | Wyndham Cove | 4.48 |
| Keppel Islands | Middle Is | Keppel Islands | Clam Bay | 6.57 |
| Keppel Islands | Clam Bay | Percy Islands | Northeast Percy | 5.94 |
| Keppel Islands | Clam Bay | Percy Islands | South Percy | 5.1 |
| Keppel Islands | Clam Bay | Percy Islands | South Percy | 10.12 |
| Keppel Islands | Egg Rock | Percy Islands | Middle Percy | 4.81 |
| … |  |  |  |  |
|  |  |  |  |  |

*(Table S2.2 continued)*

| **SOURCE** |  | **DESTINATION** |  |  |
| --- | --- | --- | --- | --- |
| **Region** | **Reef** | **Region** | **Reef** | **LOD** |
| Keppel Islands | Egg Rock | Percy Islands | Middle Percy | 5.27 |
| Keppel Islands | Egg Rock | Percy Islands | South Percy | 7.01 |
| Keppel Islands | Egg Rock | Percy Islands | South Percy | 7.97 |
| Keppel Islands | Egg Rock | Percy Islands | South Percy | 8.24 |
| Keppel Islands | Egg Rock | Percy Islands | South Percy | 12.04 |
| Keppel Islands | Halfway | Percy Islands | South Percy | 4.64 |
| Keppel Islands | Halfway | Percy Islands | South Percy | 4.69 |
| Keppel Islands | Halfway | Percy Islands | South Percy | 5.36 |
| Keppel Islands | Middle Is | Percy Islands | Northeast Percy | 5.67 |
| Keppel Islands | North Keppel East | Percy Islands | Northeast Percy | 5.58 |
| Percy Islands | South Percy | Capricorn Bunkers | Erskine | 5.65 |
| Percy Islands | South Percy | Capricorn Bunkers | Polmaise | 4.05 |
| Percy Islands | South Percy | Keppel Islands | Clam Bay | 5.5 |
| Percy Islands | South Percy | Keppel Islands | Clam Bay | 6.09 |
| Percy Islands | South Percy | Keppel Islands | Halfway | 7.15 |
| Percy Islands | South Percy | Keppel Islands | Halfway | 13.22 |
| Percy Islands | South Percy | Keppel Islands | Hummocky Is | 6.23 |
| Percy Islands | South Percy | Keppel Islands | North Keppel | 8.2 |
| Percy Islands | South Percy | Percy Islands | South Percy | 5.23 |
| Percy Islands | South Percy | Percy Islands | South Percy | 5.3 |
| Percy Islands | South Percy | Percy Islands | South Percy | 11.33 |
|  |  |  |  |  |

**REFERENCES**

1. Mapstone BD, Ayling AM, Choat JH (1998) Habitat, cross shelf and regional patterns in the distributions and abundances of some coral reef organisms on the northern Great Barrier Reef, with comment on the implications for future monitoring. In: *Research publications series no. 48*, p. 77. Great Barrier Reef Marine Park Authority, Townsville.

2. Sadovy de Mitcheson Y, Craig MT, Bertoncini AA, Carpenter KE, Cheung WWL, Choat JH, Cornish AS, Fennessy ST, Ferreira BP, Heemstra PC, Liu M, Myers RF, Pollard DA, Rhodes KL, Rocha LA, Russell BC, Samoilys MA, Sanciangco J (2013) Fishing groupers towards extinction: a global assessment of threats and extinction risks in a billion dollar fishery. *Fish and Fisheries*, **14**, 119-136.

3. Williamson DH, Russ GR, Ayling AM (2004) No-take marine reserves increase abundance and biomass of reef fish on inshore fringing reefs of the Great Barrier Reef. *Environ. Conserv.*, **31**, 149-159.

4. Emslie MJ, Logan M, Williamson DH, Ayling AM, MacNeil MA, Ceccarelli DM, Cheal AJ, Evans RD, Johns KA, Jonker MJ, Miller IR, Osborne K, Russ GR, Sweatman HPA (2015) Expectations and outcomes of reserve network performance following re-zoning of the Great Barrier Reef Marine Park. *Current Biology*, **25**, 983-992.

5. Samoilys MA, Squire LC (1994) Preliminary observations on the spawning behavior of coral trout, *Plectropomus leopardus* (Pisces: Serranidae), on the Great Barrier Reef. *Bulletin of Marine Science,* **54**, 332-342.

6. De Mitcheson YS, Cornish A, Domeier M, Colin PL, Russell M, Lindeman KC (2008) A global baseline for spawning aggregations of reef fishes. *Conservation Biology*, **22**, 1233-1244.

7. Samoilys MA (1997) Periodicity of spawning aggregations of coral trout Plectropomus leopardus (Pisces: Serranidae) on the northern Great Barrier Reef. *Marine Ecology Progress Series*, **160**, 149-159.

8. Zeller DC (1997) Home range and activity patterns of the coral trout *Plectropomus leopardus* (Serranidae). *Marine Ecology Progress Series*, **154**, 65-77.

9. Zeller DC (1998) Spawning aggregations: patterns of movement of the coral trout *Plectropomus leopardus* (Serranidae) as determined by ultrasonic telemetry. *Marine Ecology Progress Series*, **162**, 253-263.

10. Bunt CM, Kingsford MJ (2014) Movement, habitat utilization and behaviour of coral trout *Plectropomus leopardus* during and after the reproductive period on the southern Great Barrier Reef. *Marine Ecology Progress Series*, **496**, 33-45.

11. Williamson DH, Harrison HB, Almany GR, Berumen ML, Bode M, Bonin MC, Choukroun S, Doherty PJ, Frisch AJ, Saenz-Agudelo P, Jones GP (2016) Large-scale, multi-directional larval connectivity among coral reef fish populations in the Great Barrier Reef Marine Park. *Molecular Ecology,* **25**, 6039-6054.

12. Harrison HB, Feldheim KA, Jones GP, Ma K, Mansour H, Perumal S, Williamson DH, Berumen ML (2014) Validation of microsatellite multiplexes for parentage analysis and species discrimination in two hybridizing species of coral reef fish (*Plectropomus* spp., Serranidae). *Ecology and Evolution*, **4**, 2046-2057.

13. Marshall TC, Slate J, Kruuk LEB, Pemberton JM (1998) Statistical confidence for likelihood-based paternity inference in natural populations. *Molecular Ecology*, **7**, 639-655.

14. Gerber S, Chabrier P, Kremer A (2003) FAMOZ: a software for parentage analysis using dominant, codominant and uniparentally inherited markers. *Molecular Ecology Notes*, **3**, 479-481.
