# Supplementary material for: Successful validation of a larval dispersal model using genetic parentage data
Source: PLoS Biol. 2019 Jul 12;17(7):e3000380. doi: 10.1371/journal.pbio.3000380 (PMC6655847; doi:10.1371/journal.pbio.3000380)
Supplement: S2 Text — (DOCX) [file pbio.3000380.s002.docx]

**Supporting Information Text 2: Biophysical model**

**Introduction**

In this section, we describe a biophysical dispersal model of *Plectropomus maculatus* larvae in the southern Great Barrier Reef, Australia (the “GBR”). The “GBR-Larvo” model integrates adult spawning and larval behaviours with a realistic description of ocean currents. In the sections below, each element of our model is described in turn.

Our central goals in creating this model were to (1) Capture the important oceanographic conditions and dynamics during the observed dispersal events a high spatial and temporal resolution; (2) simulate larval behaviour that matches the target species, based on empirical laboratory and field measurements of that behaviour; and (3) incorporate the variation in larval behaviour that has been observed between different larvae, and also within an individual larva’s pelagic dispersal phase, including temporal stochasticity and ontogenetic variation.

**Hydrodynamic model**

LOCATION

The GBR is a complex system of over 2000 reefs and shoals on the continental shelf of eastern Queensland, stretching from 10°S to 24°S, and in many locations extending from the shelf break to the mainland coast (Figure S2.1). The goal was to model larval dispersal in the region where the genetic parentage dataset was sampled (Figure 1 in the main text; Figure S2.1), which centres on the inshore and mid-shelf reefs in the southern GBR. Specifically, the genetic parentage data was obtained from reefs belonging to GBRMPA bioregions RE8 (Coastal southern fringing reefs), RCB1 (Capricorn Bunker outer reefs), and RCB2 (Capricorn Bunker mid-shelf reefs).

The Keppel and Percy Island groups are archipelagos of high continental islands surrounded by fringing coral reefs, while the Capricorn Bunker group comprises emergent platform reefs located on the outer margin of the narrow continental shelf. The vast majority of the seafloor surrounding the three focal reef clusters is dominated by open sand habitat; apart from several deep-water shoals to the north of the Capricorn Bunkers, there are no other significant coral reef habitats within the study domain. The largest amount of reef habitat in the broader region is the Swains reefs, a dense assemblage of relatively small reefs on the far side of the Capricorn Channel (Figure S2.1). These reefs play an important hydrodynamic role, sheltering the midshelf and inshore regions from oceanic influences.

At the Keppel Islands, fringing coral reefs cover approximately 700 ha, of which 196 ha (~ 28%) is protected within a network of no-take marine reserves. The Percy Islands are surrounded by approximately 1870 ha of fringing coral reefs. The Capricorn Bunker group is comprised of a vast area of platform reefs, many of which have extensive reef flat and lagoon habitats. This study focused on eleven reefs in the northern section of the Capricorn Bunkers, bound by Polmaise Reef (southwest), Northwest Reef (northwest), North Reef (northeast), and One Tree Reef (southeast). The eleven focal reefs have a total reef area of nearly 25,700 ha, of which approximately 14,700 ha (~ 57 %) is designated as no-take marine reserve and 11,000 ha (~ 43 %) is open to fishing.

The reef map was based on two datasets: digital shape files provided by the Great Barrier Reef Marine Park Authority (GBRMPA) and habitat mapping derived from satellite imagery of 5 reef groups (Whitsunday Islands, Keppel Islands, Northern Capricorn Bunkers and Percy Islands). Bathymetry was obtained from digitised Australian Hydrographic Office charts and LASER Airborne Depth Sounder (LADS Corporation Ltd). Depths within the GBR are relatively shallow: few areas are >100m, and depths are generally <50 m north of about 17°S.

*
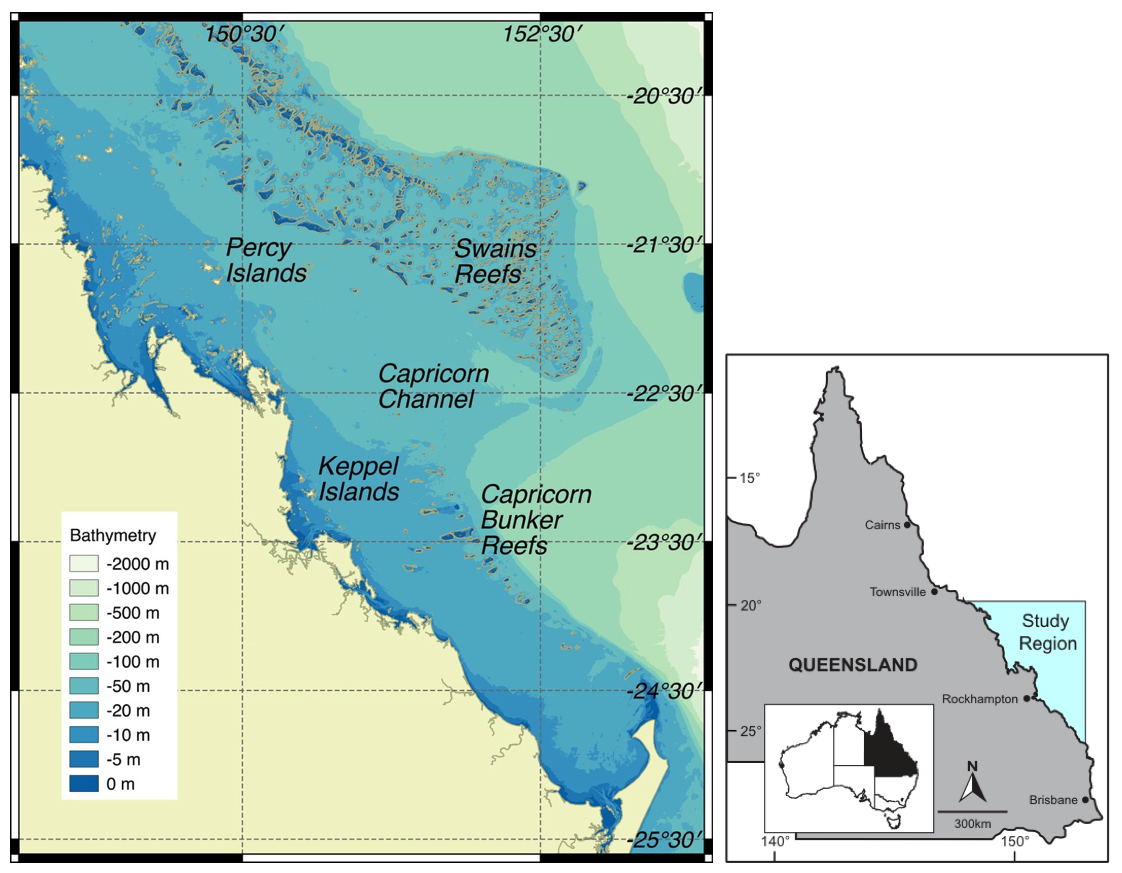
*

*Figure S2.1: Map of the study region bathymetry with key reef groups labelled (source: Etopo 2). Important features include the two distinct reef groups (midshelf and inshore reefs in the Capricorn Bunker group, the Keppel Islands group, and the Percy Islands group; offshore reefs in the Swains Reefs), and the Capricorn Channel.*


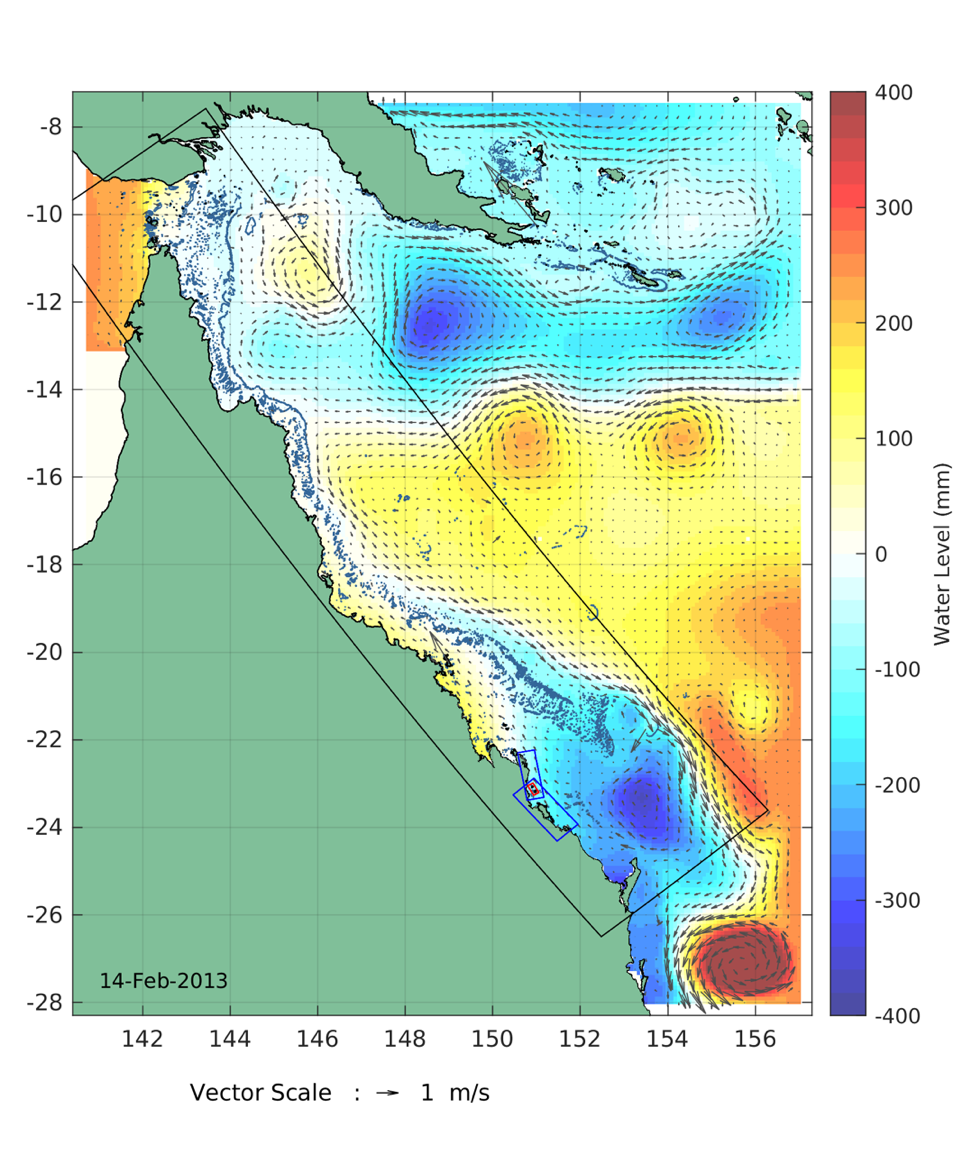


*Figure S2.2: Map of the nested hydrodynamic model domains. A model of the whole Coral Sea was used to parameterise the East Australia Current (EAC), and was forced by water height data from Weipa and Mooloolaba in Australia, Lae in Papua New Guinea, Honiara in the Solomon Islands, Port Villa in Vanuatu, and Noumea in New Caledonia. The black box shows the extent of the 1.85km/1hr model; blue boxes show the extent of the 370m/30min model; and the red box shows the 70m/15min model. Colours indicate water height, and arrows indicate water flow vectors, as predicted by the broadest-scale model, which was used to drive the EAC boundary conditions in the higher-resolution models.*

NUMERICAL MODEL

The hydrodynamic model is solved using the numerical scheme developed in James, Armsworth [1] and Luick, Mason [2]. The results of both were validated with data from current meters. Currents in the GBR are forced by tides (The modelled tidal constituents were the 2Q1, Q1, O1, NO1, P1, S1, K1, φ1, J1, OO1, UPS1, EPS2, 2N2, µ2, N2, ν2, M2, L2, T2, S2, K2 and η2), wind and oceanic influences of the seasonally varying East Australian. The model employs a version of the finite difference hydrodynamic model of Bode and Mason [3] [4]. It also incorporates a reef parameterisation scheme [5] that allows the dynamical effects of the reef matrix to be assimilated into flow simulations without requiring the reefs themselves to be fully-resolved by the model grid (which would require a prohibitively small grid size).

The hydrodynamic model’s computational grid is based on the Arakawa “C” grid [6]. Bottom friction terms (e.g., $Cdu^{2}$) decrease to very small values in water depths greater than 70 m in order to simulate the effect of stratification, which tends to isolate the surface circulation from deeper flow. Model runs are forced by wind data from the ERA-Interim CMWind Field. These data were supplied on a horizontal grid with 0.75° resolution and a 6 h time interval. The model is built from computational nested grids at four spatial resolutions (Figure S2.2). The low resolution grid, 5’ (~9 km), encompasses the whole Coral Sea and the Gulf of Carpentaria, and provides dynamic boundary conditions for the Great Barrier modelling domain (see below). The medium resolution grid is for the entire GBR, at 1' (1.85 km) (indicated by the black box in Figure S2.2). The high resolution grids encompasses the broad survey regions, and is at 0.2' (370 m) (blue boxes, Figure 2.2). The very high resolution grid focuses on the high-density complex of reefs in the Keppel Islands, and is at 0.04' (74 m) (red box, Figure 2.2).

This Coral Sea model is forced at the surface by wind stress, and at the outer boundary by average annual sea level signals synthesised from coastal and island tide gauges (Figure S2.2). A false bottom is placed at a depth of 300m to reduce topographic steering of the barotropic flow. The Coral Sea model is not intended to resolve the details of the EAC seaward of the GBR (which is a highly baroclinic domain), since larvae do not disperse within this outer model domain. Instead, its purpose is to simulate the effect of Coral Sea flows on shallow shelf currents by producing the necessary cross-shelf surface pressure gradients [7].

The GBR model and subsequent nested models use a sigma transformation in the vertical, where layers represent a fixed proportion of the water column. There are six unequally spaced vertical layers that are refined near the surface and bottom. A level 2 ½ [8] turbulence closure model is incorporated to provide vertical eddy viscosities and length scales of vertical diffusivity. Density stratification (baroclinicity) is not included as a driving force. The computed vertical diffusivity is used to determine the vertical scale of the random walk process applied to model larvae. The model is not specifically validated for flows in the study region of the southern GBR. However, it provides an accurate match to depth-averaged currents in the tidally-dominated Capricorn Channel (Figure S2.3). The ability of the model to capture the along shore low frequency flows is shown in Figure S2.4 for the long term Yongala mooring located mid-way along the GBR lagoon. The seasonal and the shorter weather-driven flows shown Figure S2.4 are the primary drivers for larval dispersal over longer distances, although flow reversals over a 1 to 2 week period can sometimes return larvae to the vicinity of their origin. The model has more difficulty recreating flow directions along an east-west axis, which could affects its ability to predict inter-regional dispersal to the Capricorn Bunkers, which lie to the east of the main Keppel Islands – Percy Islands axis (Figure S2.3 & Figure S2.4).


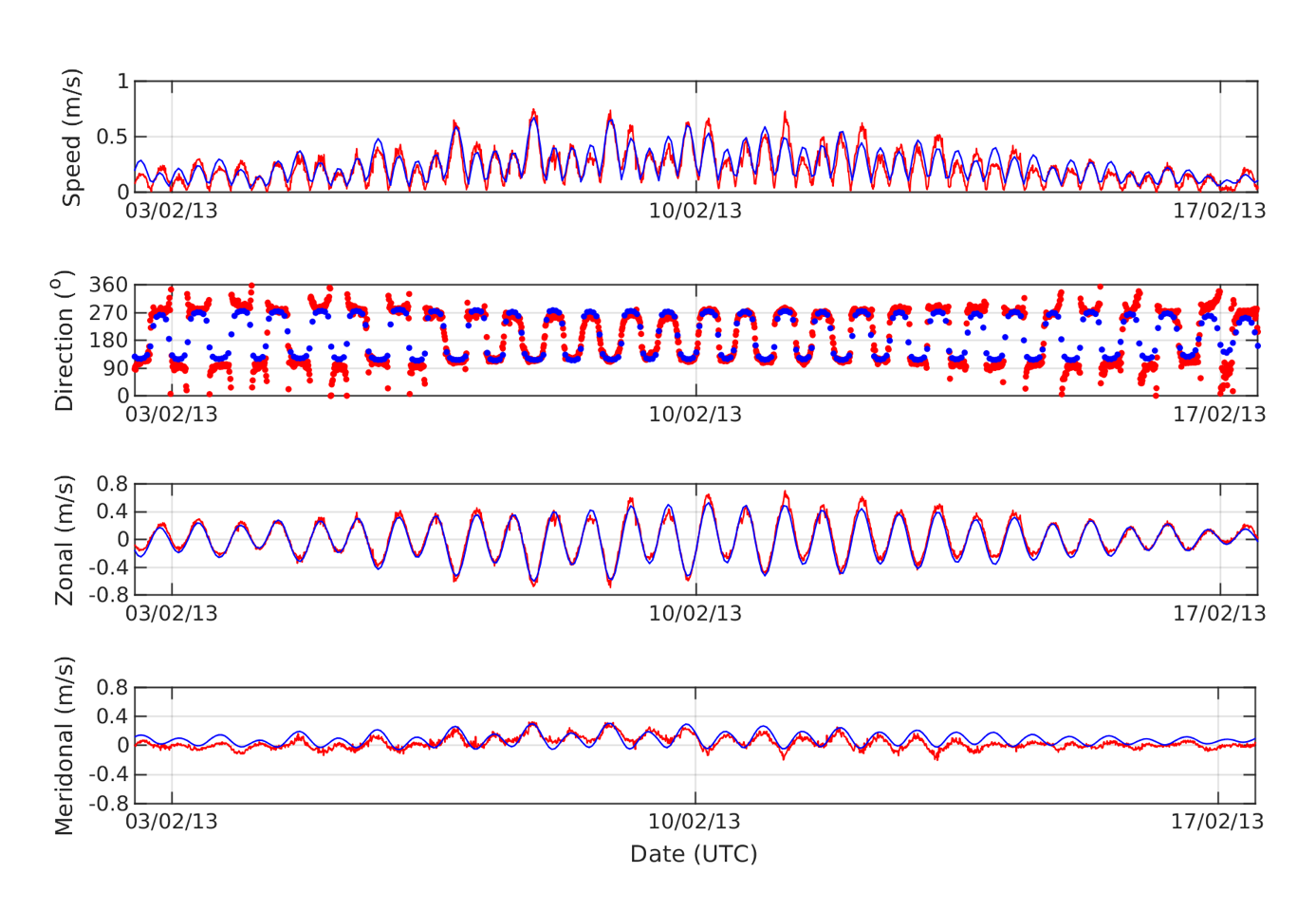


*Figure S2.3: Comparisons in depth-integrated flow between the modelled (blue) and observed data (red), in Feb 2013, for the Capricorn channel station of the Australian National Mooring Network (Lat -22.408, Lon 151.993). From top to bottom, the panels show the magnitude of the current velocity, the direction of the current, the zonal (i.e., east-west) flow, and the meridional (i.e., north-south) flow. NB: model deviation (direction) appears largest when flow magnitude is smallest (where error is least important).*

**
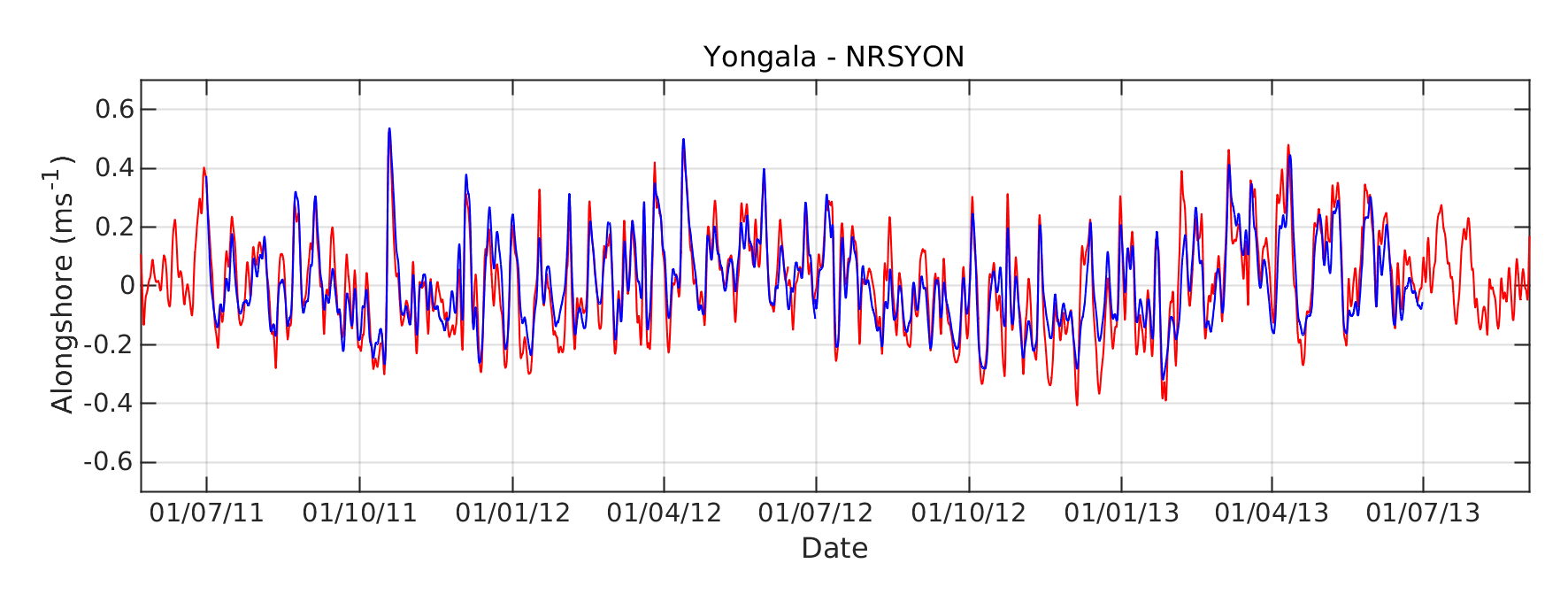
**

*Figure S2.4: Depth-integrated flow for hydrodynamic model predictions (blue) and observed data (red), between July 2011 and July 2013, for the Yongala station of the Australian National Mooring Network (Lat -19.305, Lon 147.622). The figure reveals a match to both the low-frequency annual signal of the EAC-driven flow, and the medium frequency wind-driven flows. Peaks are caused by SE winds; troughs are caused by breaks in the wind that allow the prevailing southward ocean current to dominate.*

**Biological model**

STUDY SPECIES

The genus *Plectropomus* is included in the serranid subfamily Epinephelinae, tribe Epinephelini, commonly called groupers (although *Plectropomus* species are often called coral trouts in Australia). Grouper larvae all have a similar “kite-shaped” morphology, with very long, serrate spines in both dorsal and pelvic fins [9]. When *P. maculatus* are between 4-10 mm long, the second spine of the dorsal fin is longer than the body: the pelvic-fin spine is somewhat shorter. By settlement, both spines are reduced to 1/3 of the 16-17 mm body length (standard length). Notochord flexion and formation of the caudal fin take place at a length of about 5-6 mm [9].

ADULT SPAWNING BEHAVIOUR

For the purpose of modelling larval dispersal, the most important adult behaviours to understand are the timing and location of spawning [10]. Some fishes spawn in adult habitat, while others migrate to specific, limited, often multi-species spawning aggregation sites, or over shorter distances to more diffuse locations such as reef edges [11]. To realistically model dispersal, the location where the eggs are spawned is important to determine, and the grid size of the oceanographic model must be sufficiently fine to discriminate between locations and to model local flow fields. Spawning is normally seasonal, even in the tropics, and lunar and diel periodicity in spawning are also common. This type of life history information must be included so the model propagules can be initialised at the appropriate time and location. Temporally-varying factors such as tidal currents, coastal-trapped waves and wind-influenced currents are often very important, especially in the first few days following spawning, when the eggs and larvae are closest to being passive [12, 13].

For *P. maculatus,* planktonic eggs are input at all reef edges on days surrounding new moons (± 5 days) at 1800 hrs. Approximately 300 eggs are released each day per kilometre of reef edge ($2.5\times{10}^{7}$ eggs per new moon across the system). Releases are modelled for all new moons between July 2011 and July 2013 inclusive, to span the larval spawning events observed in the genetic parentage dataset. Spawning rates are heterogeneous across this period, peaking in January each year, but this variability is implemented in post-processing (Figure S2.5). Buoyancy and diffusion parameters are applied to the pelagic eggs during incubation, then applied to vertical swimming after hatching.

Modelled full moon dates: 31/07/2011, 29/08/2011, 27/09/2011, 25/11/2011, 25/12/2011, 23/01/2012, 22/02/2012, 23/03/2012, 21/04/2012, 21/05/2012, 20/06/2012, 19/07/2012, 18/08/2012, 16/09/2012, 15/10/2012, 14/11/2012, 13/12/2012, 12/01/2013, 10/02/2013, 12/03/2013, 10/04/2013, 10/05/2013, 09/06/2013.

LARVAL BEHAVIOUR OVERVIEW

We constructed three different behavioural variants of the model, and repeated our dispersal simulations using each in turn. The first two models were plausible representations of *P. maculatus* larval behaviour, based on best empirical estimates for each of the important behavioural parameters. They differed in whether the observed variation within a population was attributable to between-individual variation or to within-individual variation. We chose these two different alternatives because the observations of larval behaviours used in the model were typically made over short time periods. The consistent model assumes that behaviour of an individual is consistent over a given ontogenetic stage lasting a few days, whereas the varying model makes no assumption about consistency of behaviour beyond the few minutes over which the behavioural observations were made.

First, a **consistent behaviour model** assumed that observed variation was attributable to between-individual differences. Each larva’s behaviour was sampled randomly from cumulative probability distribution functions (CDFs), which were defined for four different ontogenetic stages. These were defined by the larval length which was converted to age based on a linear growth assumption (see below for details). The sampled value for each parameter was maintained throughout the ontogenetic stage, and was then resampled at the beginning of each subsequent stage from its CDF. Thus in the consistent model, for example, some larvae were deep-swimmers, and others were shallow-swimmers.

Second, a **varying behaviour model** assumed that variation was attributable to within-individual variation across time. The behaviour of each larva in this model was re-sampled from the ontogenetically-appropriate CDF at each time step. In this varying model, for example, individual larvae did not occupy a particular swimming depth. Instead, each individual occupied a range of depths over the ontogenetic interval.

Thus the consistent and varying behavioural models displayed the same parameter distributions on aggregate, but explained this observed variation in different ways. For example, for swim speed, the consistent model is based on the idea that there are ‘good’ performers, as well as ‘average’ and ‘poor’ performers. In contrast, the varying model is based on the idea that performance varies with time (within the frequency distribution of observed values). While any two larvae will exhibit different behaviour at a given point in time, individuals were not different from one another on average within an ontogenetic stage. Both models were a reasonable interpretation of the observational evidence.

The final behavioural variant was a **passive behaviour model**, where larvae act as neutrally-buoyant particles with minimal behaviour.

Overall, these three behavioural variants were designed to assess the likely importance of including larval behaviour (i.e., passive vs. consistent & passive vs. varying), and the impact of different plausible behavioural assumptions (i.e., varying vs. consistent).

For dispersal modelling, the most important larval behaviours to accurately capture are vertical distribution, horizontal swimming and orientation abilities, and ability to respond to sensory cues used to achieve orientation [14] [15]. Within a particular species of fish, this behaviour can vary in a number of different ways, often by a large amount [16]; [17]. This variation comes from a range of different sources:

First, behaviour can vary on a diel basis **(Diel variation)**. This variation is most obvious in the vertical distribution of larvae, so diel influences must be included for many species.

Second, they can vary spatially **(Spatial variation)**. For example, vertical distribution is influenced by the local depth of the water column [18], and swimming speed and orientation may be location dependent. It is therefore essential to take the location of the modelled propagule into account throughout its pelagic larval duration (PLD).

Third, larvae develop morphologically during their planktonic sojourn, and so does their behaviour [19]. Late stage larvae are better swimmers (faster & with greater endurance), with superior sensory capabilities. This behaviour must therefore be understood and modelled ontogenetically [17] **(Ontogenic variation)**.

Fourth, larval behaviour varies among individuals **(Among individual variation)**. Many dispersal models only incorporate the mean values for different behaviours. For example, dispersal models commonly position larvae at a single depth, representing the centre of mass of the observed vertical distributions. However, larvae are seldom, if ever, clustered tightly at a particular depth [18, 20]. Such mean field assumptions assume that whatever influence vertical distribution has on dispersal applies equally to all individuals. Current velocity varies with depth, and the variance in vertical distribution results in different larvae being exposed to different current velocities. In short, one can’t realistically model dispersal by using average behavioural values in a system where physical and biological inputs vary spatially or temporally.

Fifth, the behaviour of a single larva can vary across time, even within the same ontogenetic stage **(Stochastic variation)**. This particular type of variation occurs much more rapidly than either diel or ontogenetic variation (i.e., on a timescale of less than an hour).


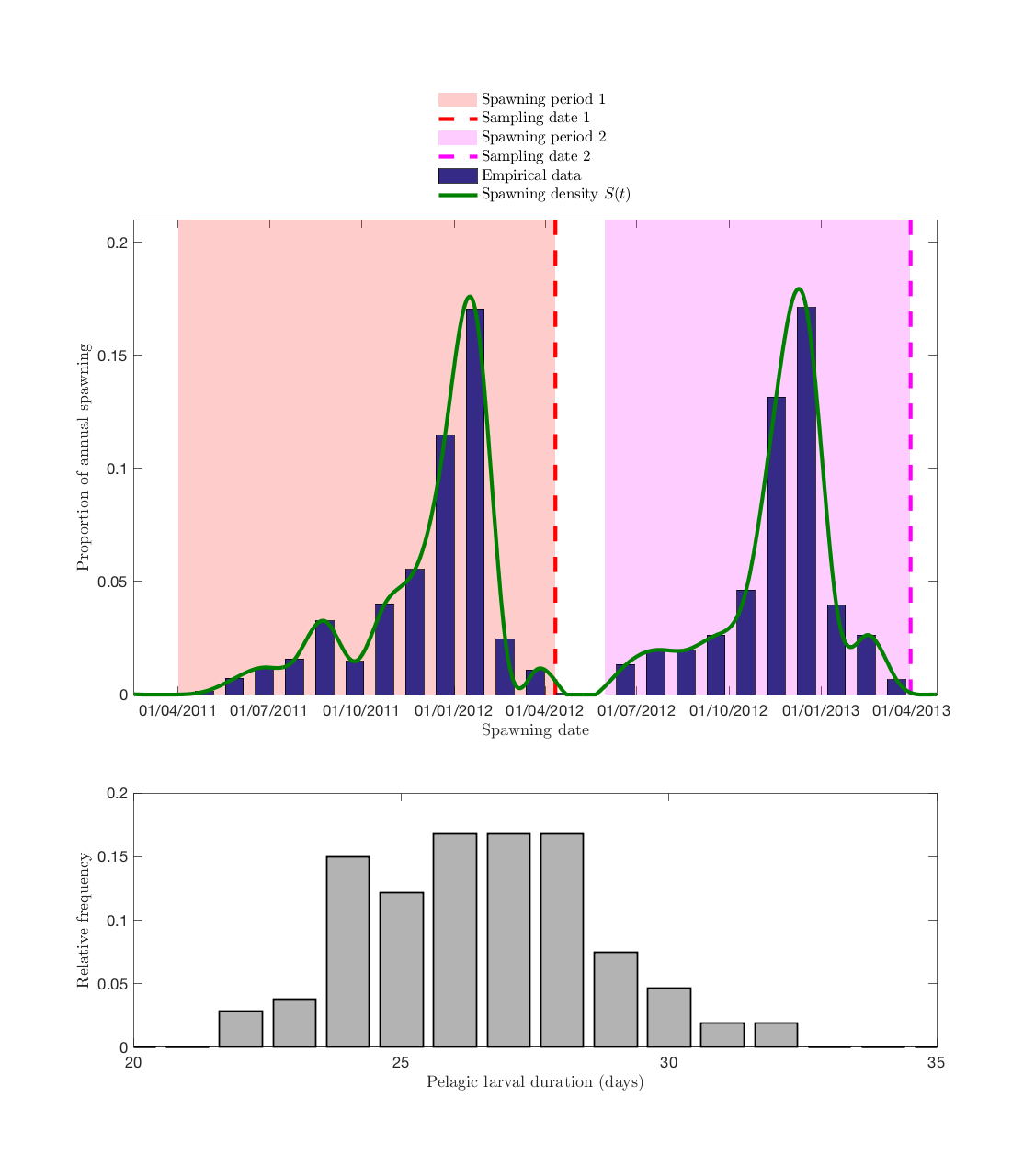


*Figure S2.5: Observed variation in the* P. maculatus *populations of the southern GBR.* ***Upper panel*** *shows the spawning density of the adults indicated by the juvenile otolith data, throughout the two sampled years. Blue bars indicate the observed data; green lines show a best-fit cubic spline, shaded regions denote the spawning season; dashed lines indicate the start dates for the juvenile sampling (each trip lasted several weeks).* ***Lower panel*** *shows a frequency distribution of pelagic larval durations for a subsample of 107* P. maculatus *juveniles from the genetic parentage study (Supporting Information Text 1). The PLD length was calculated using otolith analysis, and revealed a normally distributed set of PLDs (p=0.31, Kolmogorov-Smirnov test) with a mean of 26.5 days, with a standard deviation of 2.2 days. .*

This multidimensional variation affects the patterns of larval dispersal and settlement, the amount of variation in these patterns, and the condition and the survival of larvae until they reach their settlement reef [21, 22]. Throughout our model these sources of variation are included wherever feasible, not only to avoid bias in the predicted patterns, but also to avoid unrealistically constraining predicted dispersal outcomes [17]; [15].

To include each source of variation, cumulative distribution functions (CDFs) were created from empirical larval behaviour data (e.g., swim speed, or vertical position). These CDFs often depend on the age and location of the larvae. For example, the vertical position of a larva depends on its ontogenetic stage, the time of day, and the depth of the local water column. Or, the swimming behaviour of a larva depends on whether it is close to settlement habitat, and its ontogenetic stage. Each individual was randomly assigned a position in the CDF for each behaviour, and this behaviour was re-allocated at particular intervals (Irisson et al., 2009b). In the “consistent” model, for example, each larva’s vertical position is re-calculated at the beginning of each ontogenetic interval. In the sections that follow, each of the behavioural assumptions made for *P. maculatus* are detailed.

PELAGIC LARVAL DURATION

*Sources of variation: among individual.*

*P. maculatus* larvae that successfully settled in the southern GBR genetic parentage dataset were observed to have an average PLD of 26.5 (± 2.2 s.d.) days (Figure S2.5). About 75% of larvae settled within 24 to 29 days of hatching presumably due to the large amount of available settlement in the GBR lagoon. The larvae may have spent longer in the pelagic environment if there had not been nearby reefs upon which to settle. In the model, we assumed that larvae reach competence for settlement after 22 days dispersing, and are removed from the model if they haven’t settled within 33 days. The observed PLD in the model will therefore be an emergent property of the larval dispersal dynamics, constrained by these bounds.

VERTICAL DISTRIBUTION

*Sources of variation: diel; ontogenetic; spatial; among individual.*

Pelagic eggs are initially introduced into the water column at spawning aggregation locations, in the middle of the water column. The vertical position of pelagic eggs is thereafter determined by a combination of buoyancy and vertical diffusion in the water column, calculated using a random walk. The egg’s positive buoyancy value (Table S2.1, derived from literature sources in Table S2.2) moves the eggs towards the surface, but vertical turbulence (implemented using the diffusivity parameter from the hydrodynamic model) can move them either upward or downward (Table S2.1). The vertical distribution of the eggs is therefore an emergent property of the model, although it is noteworthy that the eggs hatch within 27 hours of spawning (Masuma et al 1996).

*Table S2.1: Egg buoyancy and vertical diffusion as discrete functions of age*

| Age (days) | Egg buoyancy (m s^-1^) | Vertical diffusion (m^2^ s^-1^) |
| --- | --- | --- |
| 0 | 0.00149 | 1.0 |
| 1.0833 | 0.00149 | 1.0 |
| > 1.0833 | 0 | 0 |

Once larvae became ‘active’ (i.e., when the eggs hatch, an **ontogenetic** change) vertical velocities (vertical diffusion) and buoyancy were ignored (except in the passive model, see below). A CDF of vertical distribution was constructed from empirical data, including plankton-net and light-trap studies and diver observation of larvae *in situ* (see Table S2.2 for literature sources). These distributions are sampled randomly for a starting value for each individual, and this is reallocated at the beginning of each ontogenetic phase (“consistent” behavioural model), or each timestep (“varying” behavioural model). As larvae age, their depth distribution continues to change. Like many species of fish, the larvae of *P. maculatus* have different distributions during the day and night (**diel** variation).

To incorporate **spatial** variation, the CDF is stretched or compressed to fit the local water column depth as follows ([18]. Where the water depth is less than 10 m, the floor is decreased to half of the full depth (Case 1, Figure S2.6). Between 10 and 20 m water column depth, the floor lowers linearly with depth from half the water column depth to its full depth (Case 2). In water columns between 20 and 35 m deep, the floor is the full depth (Case 3). For water depth greater than 35 m, the floor is kept at 35m (Case 4).

For the consistent larval behavioural model, the location of a larva in the water column is randomly allocated from the CDF at the beginning of each ontogenetic stage (**among individual** variation). For the varying model, this depth value is randomly allocated each timestep. For the passive mode, larvae are assumed to follow the same vertical distribution dynamics as the passive eggs, although they have a different buoyancy.

ORIENTATION, SETTLEMENT & INTERACTION WITH LAND

*Sources of variation: ontogenetic; spatial; among individual.*

Larval orientation is modelled using two parameters. The first is $0\leq\theta<2\pi$, which describes the angle of an individual’s mean orientation, with $\theta=0$ indicating a northward bearing. The second parameter, $0\leq r\leq1$, measures the precision of the larva’s orientation as measured over the observation period (**stochastic** variation). A value of $r=0$ corresponds to a uniform distribution of bearings – that is, a larva that moves in all directions with equal probability. A value of $r=1$ indicates the opposite – that the larva consistently travels in the $\theta$–direction [23].


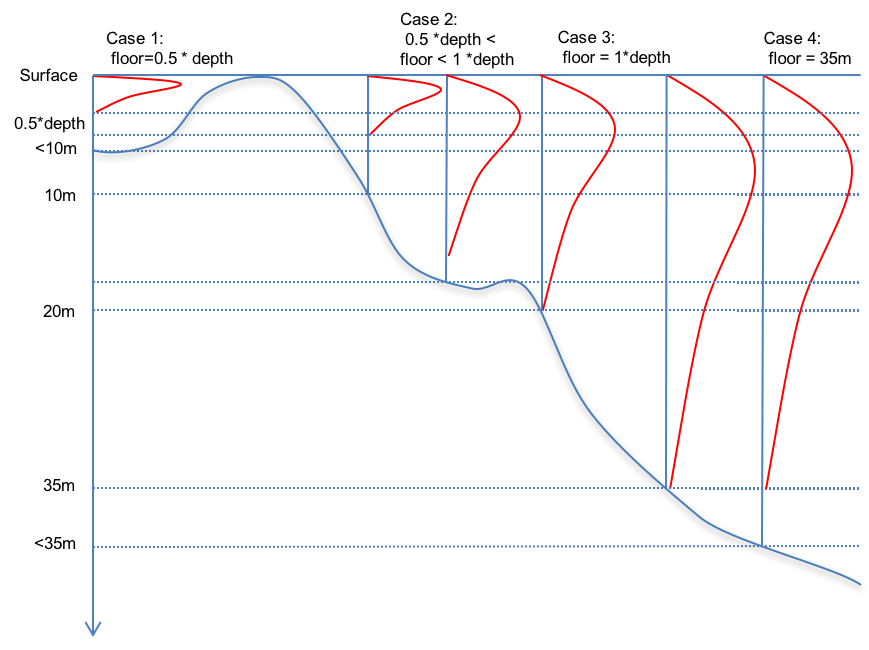


*Figure S2.6: Vertical distribution of larvae in the water column, as a function of local water depth. In water shallower than 10m (Case 1), the “floor” of the distribution (the maximum larval depth) is located halfway to the seafloor. In water between 10 m and 20 m deep (Case 2), the floor linearly increases from halfway to the full depth. Between 20 m and 35m (Case 3), the CDF floor is the seafloor. Beyond a depth of 35m, the CDF floor remains at 35m.*

Each larva’s $\left( r,\theta\right)$ values change through its development (**ontogenic** variation). During the egg phase the larva do not actively orient themselves or swim. Once they become active, each larva is randomly allocated a particular $\left( r,\theta\right)$ pair from two independent CDFs, which vary with ontogenetic stage (see Table S2.2 for the literature sources used to parameterise the CDFs). For *P. maculatus*, these orientation CDFs come from diver observation of larvae *in situ*. For the consistent larval behavioural model, values are randomly allocated at the beginning of each ontogenetic stage (**among individual** variation). For the varying model, values are randomly allocated each timestep.

For the two active swimming models (the consistent model and the varying model), important **ontogenic** variation occurs at the point of larval competency (i.e., the age where a larva becomes developed enough to settle on demersal habitat: in the case of *P. maculatus*, this is assumed to be at the shortest PLD, i.e., 24 days old). Before the onset of competency, each individual larva’s $\left( r,\theta\right)$ values are location-independent, as larvae are not responding to spatial cues. However, once they are competent to settle, larvae orient themselves towards the nearest reef that is within their detection radius (**spatial** variation). Settlement-stage larvae can swim toward and locate a sound source *in situ* [24]; [25], and laboratory measurements show that larvae of reef fishes as small as 8 mm can hear [26]. The distance from which settlement-stage larvae can detect a reef sound source was determined from laboratory studies [26, 27] and published measurements of sound propagation in reef waters [28-30].

In their attempt to reach the settlement habitat, larvae orientate themselves directly toward the nearest reef, without regard to water movement, rather like a pursuit curve. The radius of a species’ detection zone is based on its hearing abilities, and the propagation of reef-based sound (see [26, 31]). Typically, competent larvae are expected to be able to hear a reef from 4 km to 8 km, (but see [32] who show that these estimates may be conservative). When outside a reef detection zone, orientation is determined in the same manner as pre-competent larvae.

For the passive larval behaviour model, a 1 km settlement zone around reefs applies (following [1]). If a competent, passive larva comes within 1 km of a reef, it is assumed to settle on that reef. This “settlement zone” approach is used because truly passive larvae seldom settle if they are required to encounter a reef. Water tends to flow around reefs rather than into them, and so therefore do passive particles. Further, it is clear settlement-stage larvae have well-developed mobility and senses [19], so settlement cannot be considered a truly passive process, even if dispersal might be.

For all behavioural models, precompetent larvae that encounter a shoreline are repositioned into a randomly-chosen adjacent grid cell. This is repeated until the larva no longer encounters a shoreline. In effect, it is assumed that larvae can detect a nearby shoreline and avoid it either by alteration of vertical distribution or horizontal swimming.

GROWTH AND SIZE

*Sources of variation: ontogenic.*

No published growth rates are available for *P. maculatus* larvae so a linear growth rate $G$was calculated based on mean values for the size at hatching $H$, the size at settlement $Z$, and the mean PLD $P$:

$$G\left( \mathrm{mm} \mathrm{day}^{-1} \right)=\frac{Z-H}{P}.$$

This formula was used to convert size-based behavioural information from the literature into age-based information, thus driving all of our **ontogenetic** variation in behaviour. Larvae were allowed to settle at any time from the shortest to longest reported PLD, and removed from a model run if they hadn't settled at the end of the PLD range. This creates both **spatial** variation and **among-individual** variation, given that the realised PLD will depend on the location of the source reef and the dispersal trajectory.

SWIMMING BEHAVIOUR

*Sources of variation: ontogenic, among-individual.*

Where data on swimming speed ontogeny of *Plectropomus* larvae are lacking, it is assumed that the species will have swimming abilities comparable to larvae from species in the related grouper genus *Epinephelus*. Epinepheline serranid larvae have much lower initial increase in swimming speed with size than many other taxa [19]. This is probably due to the very large spines of the dorsal and pelvic fins present in the early larvae. As a result, larval epinepheline serranids initially have relatively limited ability to influence their dispersal by swimming horizontally. However, thereafter, the rate of increase in swim speed increases, so that by settlement, these larvae are among the fastest swimmers amongst reef-fish larvae. This likely means that physical forces will be a more important factor during the early stages of larval epinepheline serranids than in other families.

Larval swimming speed can be parameterised from diver observations of larvae *in situ* (e.g., [33], or they can be estimated from a species’ critical speed (*Ucrit*, [34], based on empirical relationships [19]). In laboratory flumes, three *Epinephelus* species had very little swimming ability until about 7 mm (sizes are standard length), shortly after caudal-fin formation, and thereafter critical speed (*sensu* [34]) increased at 2.2-2.5 cm s^-1^ per mm of growth, reaching 20-30 cm s^-1^ at 16-17 mm [35]. In two *Epinephelus* species, speed in the ocean (*in situ* speed) increased from 5-10 cm s^-1^ at 10 mm to 15-25 cm s^-1^ at 16-17 mm [33]. At settlement, a close-relative of *P. maculatus*, *Plectropomus leopardus* had 16.6 cm s^-1^ mean *in situ* speed [36]. The bright red *P. maculatus* larvae settle onto coral reefs after a PLD of at least 22 days.

These observations are implemented in the biophysical models using ontogenetic stage-specific CDFs of the swimming speed $S_{M}$ (**ontogenetic** variation, and **among-individual** variation). The consistent model allocates $S_{M}$ at the start of each ontogenetic stage; the varying model resamples the values each timestep. The passive model assumes $S_{M}=0.$ Importantly, the value of $S_{M}$ does not uniquely determine the net swimming speed, that is, $S_{N}\neq S_{M}$. As described in the Orientation section, a larva’s swimming orientation varies with time according to parameter $r$. A more variable swimming direction will result in slower net movement in the dominant direction $\theta$, and net forward progress is therefore modelled as $S_{N}=S+M r$.

Both *in situ* and laboratory-based measures of larval swimming speed are typically made over relatively brief time periods, so there may be questions about how long these speeds can be maintained. Swimming endurance of larval fishes increases with age [19], and, if fed, older larvae in the laboratory can swim nearly constantly for several days to a week or more, and still grow and begin to metamorphose [37] [38]. As larvae *in situ* feed ‘on the run’ [39], swimming endurance for larvae that have completed caudal-fin formation (typically from 5-8 mm long) is assumed to be unlimited within the confines of the PLD.

MORTALITY

*Sources of variation: spatial; among individual.*

Larval mortality is an important but uncertain quantity in marine population ecology. There are no published larval mortality estimates for the most reef-fish species, including *P. maculatus*. Therefore a range of mortality rates are used, ranging between 0 to 46% per day, as obtained from [40]. The two lower mortality rates (0 & 3% per day) provided dispersal outcomes that were somewhat different from those of the two higher rates (18 & 46% per day), but these differences had only very minor alterations to the results or conclusions of the model validation. The higher performance and goodness-of-fit of the consistent model held for all mortality rates.

Each virtual larva is tracked for the full PLD. Each particle is assumed to represent many larvae, and the proportion that are dead as a function of the observed PLD is calculated after the biophysical simulations are complete (i.e., a “cohort” modelling approach). The mortality rate for each larva is therefore subject to **spatial** variation, and to **among-individual** variation, because each larva’s PLD will depend on its source reef and dispersal trajectory.

Settlement, the period of transition between the pelagic and demersal environments, is a time of very high mortality for most demersal fishes, and particularly for coral reef fishes [41]. As a result, the estimated numbers of larvae reaching the end of their PLD approaching a reef to settle will be much greater than the numbers of larvae that successfully settle or recruit. This last means that any estimates of connectivity derived from dispersal models, including this one, that do not take settlement mortality into account can, at best, provide relative, not absolute, values of recruitment and connectivity. Fortunately, the genetic parentage data used to validate the biophysical model also measures the relative make-up of the settlement cohort. If that settlement mortality is assumed to be unaffected by the source reef location (and there is no evidence to suggest that mortality rates will differ with larval source over the relatively homogeneous habitat in the southern GBR that is modelled here), then fitting methods can be applied without considering the precise functional form of settlement mortality.

**Sources for larval behaviour and other model inputs.**

See Table S2.2 for the literature sources used to parameterise the larval behaviour elements of the model. Information on all inputs for *P. maculatus* was not always available, so information for related species from the same subfamily Epinephelinae of the family Serranidae was substituted. In the first instance, information from the very closely related congener, *Plectropomus leopardus* was used, and then for the confamilial *Epinephelus coioides* for which the most complete information was available.

The substitution of information from other species, albeit closely related, is likely to contribute error to the fit between the biophysical model and the parentage data. It rarely possible to obtain all the information required to parameterize dispersal models for particular study species. The performance of the biophysical model in spite of these substitutions is therefore encouraging.

*Table S2.2: Sources for behavioural information used to parameterize the biophysical dispersal model.*

| **Biophysical model element** | **Species or habitat** | **Reference** |
| --- | --- | --- |
| Pelagic larval duration | *Plectropomus maculatus* | [42] |
| Spawning times (seasonal, lunar, diel) | *Plectropomus maculatus* | [42] |
| Spawning locations | All inshore reefs in the southern GBR, at half density on some mid-shelf reefs in the southern GBR, and 5% abundance on offshore reefs.. | [42, 43] |
| Egg incubation time and size | *Plectropomus leopardus* | [44] |
| Egg buoyancy | Range of species | [45] |
| Size at hatch | *Plectropomus leopardus* | [44] |
| Size at settlement | *Plectropomus leopardus* | [36] |
| Vertical distribution (young larvae: plankton tows: diel) | *Plectropomus* sp | [20], and unpublished |
| Vertical distribution  (older larvae: day *in situ* observation) | *Plectropomus leopardus &*  *Epinephelus coioides* | [36]; [33] |
| Vertical distribution (older larvae, light trap: night) | *Plectropomus leopardus* &  *P. maculatus* | [46] |
| Swim speed | *Plectropomus leopardus* & *Epinephelus coioides* | [35, 36]; [33] |
| Swim orientation | *Plectropomus leopardus* & *Epinephelus coioides* | [36]; [33] |
| Sensory zone (hearing) | *Plectropomus leopardus* | [47] |
| Settlement habitat | Coral reefs | [42] |
| Mortality | Range of 42 species | [40] |

**Acknowledgements**

The construction of the model was supported by a Marine and Tropical Science Research Facility grant to Leis and Mason, by the Australian Museum and Australian Maritime College, and by an ARC Linkage Grant to GP Jones et al. Contributions to the modelling effort by M. Ashcroft and P. Flemons (Australian Museum GIS Unit), R.J. Pears and S. King (GBRMPA), and particularly M.N. Yerman (formerly, Australian Museum) are gratefully acknowledged.

**References**

1. James MK, Armsworth PR, Mason LB, Bode L. The structure of reef fish metapopulations: modelling larval dispersal and retention patterns. Proceedings of the Royal Society, London, B. 2002;269:2079-86. doi: 10.1098/rspb.2002.2128.

2. Luick JL, Mason LB, Hardy T, Furnas MJ. Circulation in the Great Barrier Reef Lagoon using numerical tracers and in situ data. Cont Shelf Res. 2007;27:757-78. doi: 10.1016/j.csr.2006.11.020.

3. Bode L, Mason LB. Application of an implicit hydrodynamic model over a range of spatial scales. In: Stewart D, Gardner H, Singleton D, editors. Computational Techniques and Applications: CTAC93: World Scientific Press; 1994. p. 112-21.

4. Bode L, Mason LB, editors. Tidal modelling in Torres Strait and the Gulf of Papua. Proceedings of PACON ‘94; 1995 1995; Townsville: James Cook University; 1995.

5. Bode L, Mason LB, Middleton JH. Reef parameterisation schemes for long wave models. Prog Oceanogr. 1997;40:285-324. doi: 10.1016/S0079-6611(98)00006-8.

6. Mesinger F, Arakawa A. Numerical methods used in atmospheric models. Global Atmospheric Research Program Publication 1976;17(1):1-64.

7. Berrisford P, Dee D, Poli P, Brugge R, Fielding M, Fuentes M, et al. ERA Report Series Interim Archive Version 2.0. Technical Report ECMWF 2011;(1):1-23.

8. Mellor GL, Yamada T. Development of a tubulence closure model for geophysical fluid problems. Reviews of Geophysics and Space Physics. 1982;20:851-75. doi: 10.1029/RG020i004p00851

9. Leis JM. Larval development of four species of the IndoPacific coral trout genus *Plectropomus* (Pisces: Serranidae: Epinephelinae) with an analysis of the relationships of the genus. Bull Mar Sci. 1986;38(3):525-52.

10. Gallego A, North EW. Initial conditions: spawning locations. In: North EW, Gallego A, Petitgas P, editors. Manual of recommended practices for modelling physical-biological interactions during fish early life ICES Cooperative Research Report No 295. Copenhagen: International Council for the Exploration of the Sea; 2009. p. 20-1.

11. Sadovy de Micheson Y, Colin PL. Reef fish spawning aggregations: biology, research and management. Heidelberg: Springer; 2012. 643 p.

12. Donahue MJ, Karnauskas M, Toews C, Paris CB. Location isn't everything: timing of spawning aggregations optimizes larval replenishment. PLOS One. 2015;10(6):e0130694. doi: 10.1371/journal.pone.0130694.

13. Kough AS, Paris CB. The influence of spawning periodicity on population connectivity. Coral Reefs. 2015;34:753-7. doi: 10.1007/s00338-015-1311-1.

14. North E, Gallego A, Petitgas P. Manual of recommended practices for modelling physical-biological interactions in fish early life. Copenhagen: International Council for the Exploration of the Sea; 2009 2009. 1-112 p.

15. Staaterman E, Paris CB. Modelling larval fish navigation: the way forward. ICES J Mar Sci. 2013;71(4):1-7. doi: 10.1093/icesjms/fst103.

16. Leis JM. Are larvae of demersal fishes plankton or nekton? Adv Mar Biol. 2006;51:59-141. doi: 10.1016/S0065-2881(06)51002-8.

17. Irisson J-O, Leis JM, Paris CB, Browman H. Biological Processes: Behavior and Settlement. In: North EW, Gallego A, Petitgas P, editors. Manual of recommended practices for modelling physical-biological interactions during fish early life. 295: International Council for the Exploration of the Sea Cooperative Research Report 295; 2009. p. 42-59.

18. Leis JM. Vertical distribution behaviour and its spatial variation in late-stage larvae of coral-reef fishes during the day. Mar Freshwat Behav Physiol. 2004;37(2):65-88. doi: 10.1080/10236240410001705761.

19. Leis JM. Ontogeny of behaviour in larvae of marine demersal fishes. Ichthyol Res. 2010;57:325-42. doi: 10.1007/s10228-010-0177-z.

20. Leis JM. Vertical distribution of fish larvae in the Great Barrier Reef Lagoon, Australia. Mar Biol. 1991;109:157-66. doi: 10.1007/BF0132024.

21. Shima JS, Swearer SE. The legacy of dispersal: larval experience shapes persistence later in the life of a reef fish. J Anim Ecol. 2010;79:1308-14. doi: 10.1111/j.1365-2656.2010.01733.x.

22. Nanninga GB, Berumen ML. The role of individual variation in marine larval dispersal. Frontiers in Marine Science. 2014;1(71):1-17. doi: 10.3389/fmars.2014.00071.

23. Zar JH. Biostatistical Analysis, 3rd ed. Upper Saddle River, N.J.: Prentice Hall; 1996 1996.

24. Simpson SD, Meekan MG, McCauley RD, Jeffs A. Attraction of settlement-stage coral reef fishes to reef noise. Mar Ecol Prog Ser. 2004;276:263-8. doi: 10.3354/meps276263.

25. Leis JM, Siebeck UE, Dixson DL. How Nemo finds home: the neuroecology of dispersal and of population connectivity in larvae of marine fishes. Integr Comp Biol. 2011;51(5):826-43. doi: 10.1093/icb/icr004.

26. Wright KJ, Higgs DM, Leis JM. Ontogenetic and interspecific variation in hearing ability in marine fish larvae. Mar Ecol Prog Ser. 2011;424:1-13. doi: 10.3354/meps09004.

27. Wright KJ, Higgs DM, Belanger AJ, Leis JM. Auditory and olfactory abilities of pre-settlement larvae and post-settlement juveniles of a coral reef damselfish (Pisces: Pomacentridae). Mar Biol. 2005;147(6):1425-34. doi: 10.1007/s00227-005-0028-z.

28. Cato DH. Marine biological choruses in tropical waters near Australia. Journal of the Acoustics Society of America. 1978;64:736-43. doi: 10.1121/1.382038.

29. Cato DH. The biological contribution to the ambient noise in waters near Australia. Acoustics Australia. 1992;20:76-80.

30. McCauley RD, Cato DH. Patterns of fish calling in a nearshore environment in the Great Barrier Reef. Philosophical Transactions of the Royal Society of London B. 2000;355:1289-93.

31. Wright KJ, Higgs DM, Cato DH, Leis JM. Auditory sensitivity in settlement-stage larvae of coral reef fishes. Coral Reefs. 2010;29(1):235-43. doi: 10.1007/s00338-009-0572-y.

32. Radford CA, Tindle CT, Montgomery JC, Jeffs AG. Modelling a reef as an extended sound source increases the predicted range at which reef noise may be heard by fish larvae Mar Ecol Prog Ser. 2011;438:167-74. doi: 10.3354/meps09312

33. Leis JM, Hay AC, Howarth GJ. Ontogeny of *in situ* behaviours relevant to dispersal and connectivity in larvae of coral-reef fishes Mar Ecol Prog Ser. 2009;379:163-79. doi: 10.3354/meps07904.

34. Brett JR. The respiratory metabolism and swimming performance of young sockeye salmon. Journal of Fisheries Research Board of Canada. 1964;21:1183-226. doi: 10.1139/f64-103.

35. Leis JM, Hay AC, Lockett MM, Chen J-P, Fang L-S. Ontogeny of swimming speed in larvae of pelagic-spawning, tropical, marine fishes. Mar Ecol Prog Ser. 2007;349:257-69. doi: 10.3354/meps07107.

36. Leis JM, Carson-Ewart BM. *In situ* swimming and settlement behaviour of larvae of an Indo-Pacific coral-reef fish, the Coral Trout (Pisces, Serranidae, *Plectropomus leopardus*). Mar Biol. 1999;134:51-64. doi: 10.1007/s002270050524.

37. Fisher R, Bellwood DR. Effects of feeding on the sustained swimming abilities of late-stage larval *Amphiprion melanopus*. Coral Reefs. 2001;20:151-4. doi: 10.1007/s003380100149.

38. Leis JM, Clark DL. Feeding greatly enhances swimming endurance of settlement-stage reef-fish larvae of damselfishes (Pomacentridae). Ichthyol Res. 2005;52(2):185-8. doi: Doi 10.1007/S10228-004-0265-Z.

39. Leis JM, Carson-Ewart BM. Complex behaviour by coral-reef fish larvae in open-water and near-reef pelagic environments. Environ Biol Fishes. 1998;53:259-66. doi: 10.1023/A:1007424719764.

40. Cowen RK, Lwiza KMM, Sponaugle S, Paris CB, Olson DB. Connectivity of marine populations: open or closed? Science. 2000;287:857-9. doi: 10.1126/science.287.5454.857.

41. Almany GR, Webster MS. The predation gauntlet: early post-settlement mortality in reef fishes. Coral Reefs. 2006;25(1):19-22. doi: 10.1007/s00338-005-0044-y.

42. Williamson D, Harrison H, Almany G, Berumen M, Bode M, Bonin M, et al. Large-scale, multidirectional larval connectivity among coral reef fish populations in the Great Barrier Reef Marine Park. Mol Ecol. 2016;25(24):6039-54. doi: 10.1111/mec.13908.

43. Samoilys MA, Squire LC. Preliminary observations on the spawning behavior of coral trout, *Plectropomus leopardus* (Pisces: Serranidae) on the Great Barrier Reef. Bull Mar Sci. 1994;54(1):333-42.

44. Masuma S, Tezuka N, K. Teruya K. Embryonic and morphological development of larval and juvenile coral trout, *Plectropomus leopardus*. Jap J Ichthyol. 1993;40(3):333-42. doi: 10.11369/jji1950.40.333.

45. Jung K-M, Folkvord A, Kjesbu OS, Agnalt AL, Thorsen A, Sundby S. Egg buoyancy variability in local populations of Atlantic cod (*Gadus morhua*). Mar Biol. 2012;159:1969-80. doi: 10.1007/s00227-012-1984-8.

46. Doherty PJ, Fowler AJ, Samoilys MA, Harris DA. Monitoring the replenishment of coral trout (Pisces: Serranidae) populations. Bull Mar Sci. 1994;54(1):343-55.

47. Wright KJ, Higgs DM, Belanger AJ, Leis JM. Auditory and olfactory abilities of larvae of the Indo-Pacific Coral Trout *Plectropomus leopardus* (Lacepède) at settlement. J Fish Biol. 2008;72:2543-56. doi: 10.1111/j.1095-8649.2008.01864.x.
