# Supplementary material for: Successful validation of a larval dispersal model using genetic parentage data
Source: PLoS Biol. 2019 Jul 12;17(7):e3000380. doi: 10.1371/journal.pbio.3000380 (PMC6655847; doi:10.1371/journal.pbio.3000380)
Supplement: S3 Text — (DOCX) [file pbio.3000380.s003.docx]

**Supporting Information Text 3: Validation methods**


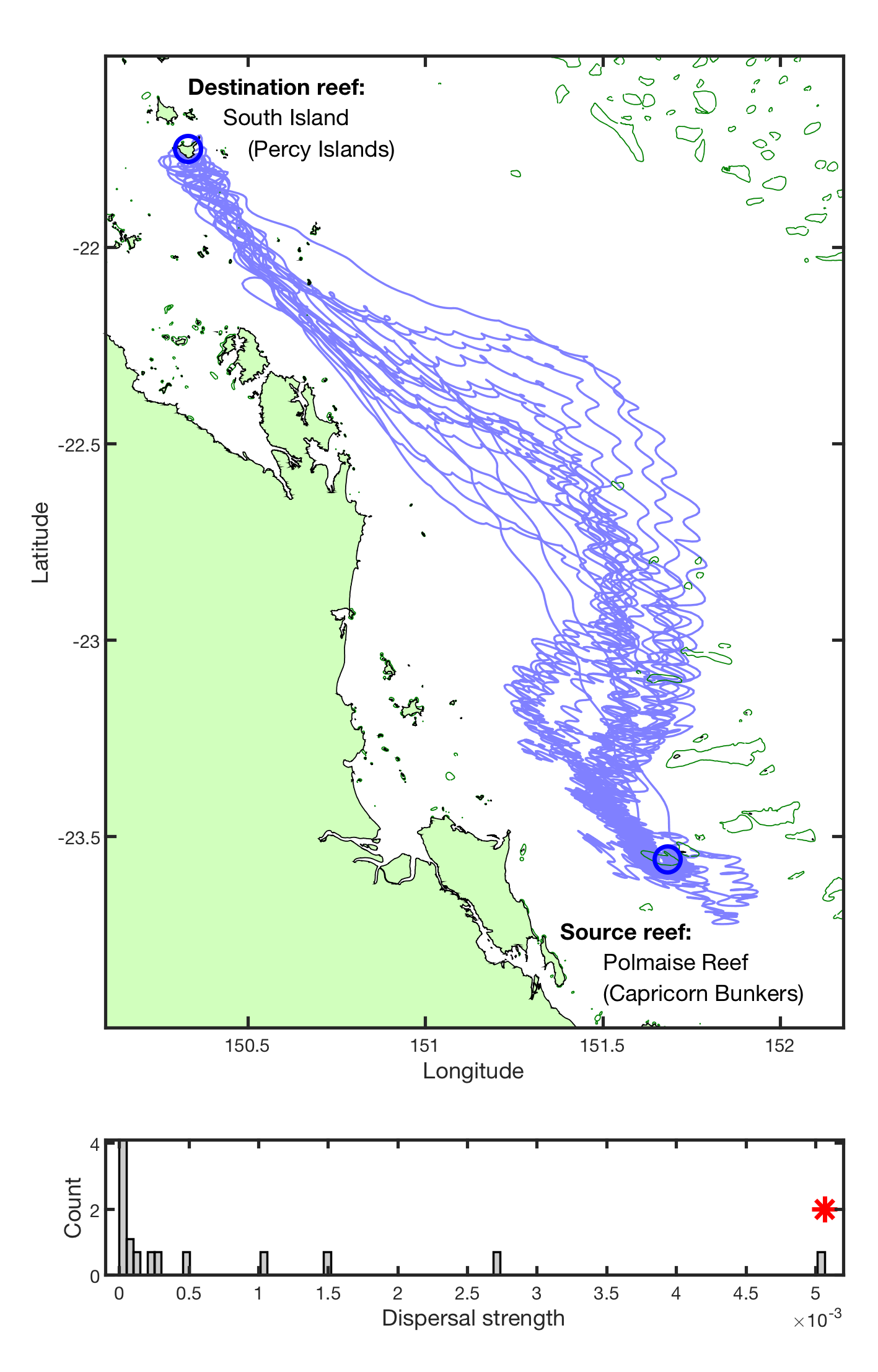


*Fig S3.1:* ***Upper panel*** *shows multiple dispersal trajectories that link Polmaise Reef in the Capricorn Bunker group to South Island in the Percy Island group, generated by the consistent larval behavioural model. Larvae in the genetic parentage dataset were also observed to connect these two reefs. Green lines show reef outlines; blue lines track the location of multiple larvae following spawning on 25/11/2011.* ***Lower panel****. The red star* *shows the strength of the connection from Polmaise to South Island according to the consistent biophysical model. These reefs are 230 km apart. The grey bars show the frequency distribution of connections between all reefs in the southern Great Barrier Reef that are between 220-240 km apart, according to the consistent model. The observed connection is the strongest in the system over that distance (note that the grey bar at* $5\times{10}^{-3}$ *is the Polmaise Reef-South Island connection). In this panel, dispersal strength is the probability that a larva spawned at reef* i *will disperse, survive and settle on reef* j*.*

**Event matching**

Our first validation comparison tests whether the specific dispersal events observed in the genetic parentage dataset are also present in the biophysical model simulations. For a successful event match, we require the biophysical model to simulate a dispersal event with the same source reef, destination reef, and approximate spawning time as a given parentage assignment. As an example, Fig S3.1 shows multiple simulated biophysical model trajectories (from the *“*consistent” model) that match the observed dispersal event from Polmaise Reef in the Capricorn Bunker group, to South Island in the Percy Island group.

The genetic parentage dataset includes 69 positive parentage assignments like the one between Polmaise Reef and South Island. For each of these assignments, we identified the new moon that was closest to the estimated spawning date of the sampled juvenile, as measured by otolith analysis. We also included the new moon immediately before and after the estimated spawning time, to account for uncertainty in either the otolith dating, or our assumption about *Plectropomus maculatus’s* new moon spawning behaviour. We then determined whether any simulated larvae from those spawning events left the source reef and dispersed to the destination reef. For the consistent biophysical model, 66 of the 69 larval dispersal events (96%) that were assigned in the parentage dataset were also simulated by the biophysical model at the appropriate location and time. The remaining 3 dispersal events did occur in the consistent model, but at different times – always within 2 full moons of the otolith-defined date.

This high level of matching was not observed for either of the other two biophysical models. For the varying model, only 32 (47%) of the observed dispersal events could be spatiotemporally matched, while for the passive model, only 34 (49%) were also found in the simulations.


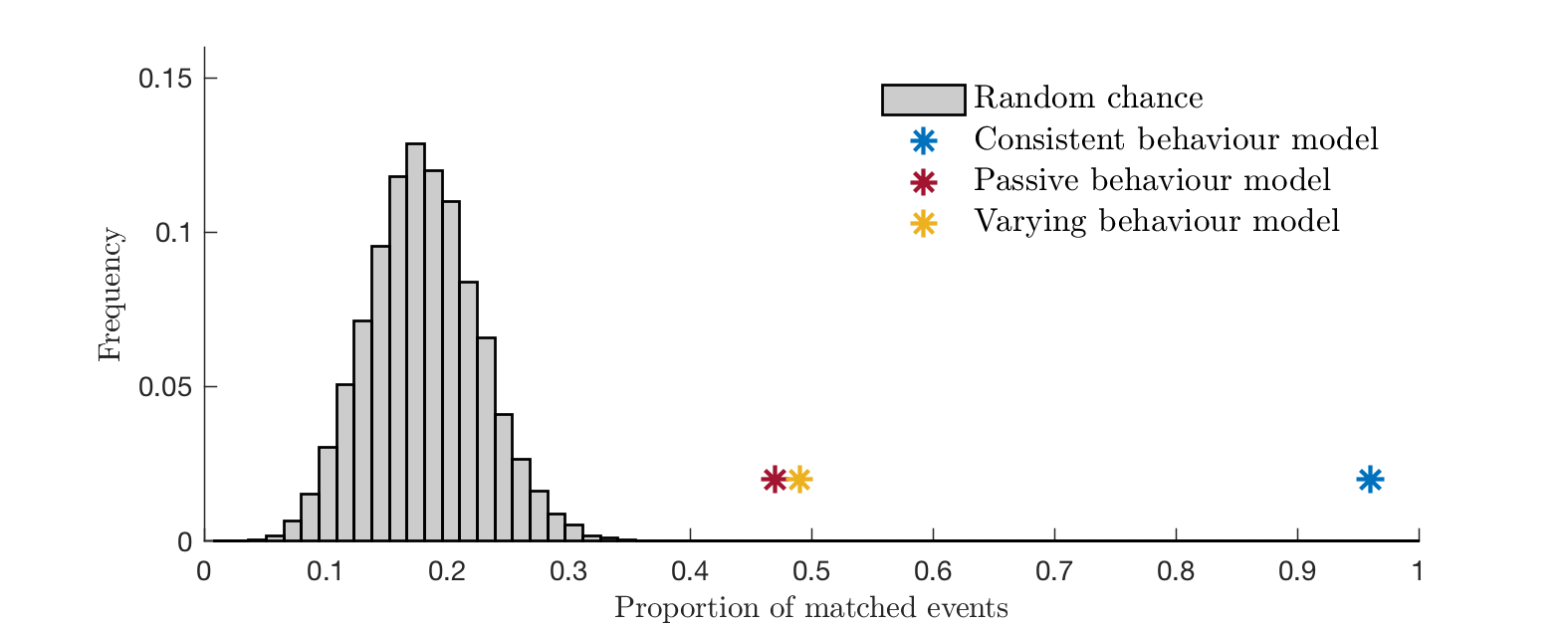


*Fig S3.2: The observed number of event matches between the biophysical models and the parentage dataset is very unlikely by random chance. The coloured markers indicate the proportion of dispersal events observed in the parentage dataset that were recreated by each of the three biophysical models. The gray bars indicate the proportion of matches expected by random chance (simulated 10^5^ times).*

These outcomes are unlikely to have occurred by random chance – particularly for the consistent model. Most (83%) reef-to-reef connections in the southern Great Barrier Reef (GBR) do not occur at each spawning event modelled by the consistent model. If the observed parentage assignment dataset were generated by a completely different process, the probability that any particular assignment would be found by chance to have occurred in the consistent model is therefore 0.17. The expected proportion of matching events is therefore shown by the histogram in Fig S3.2, and the overall probability that we would randomly find the observed number of matches is lower than $p={10}^{-6}$.


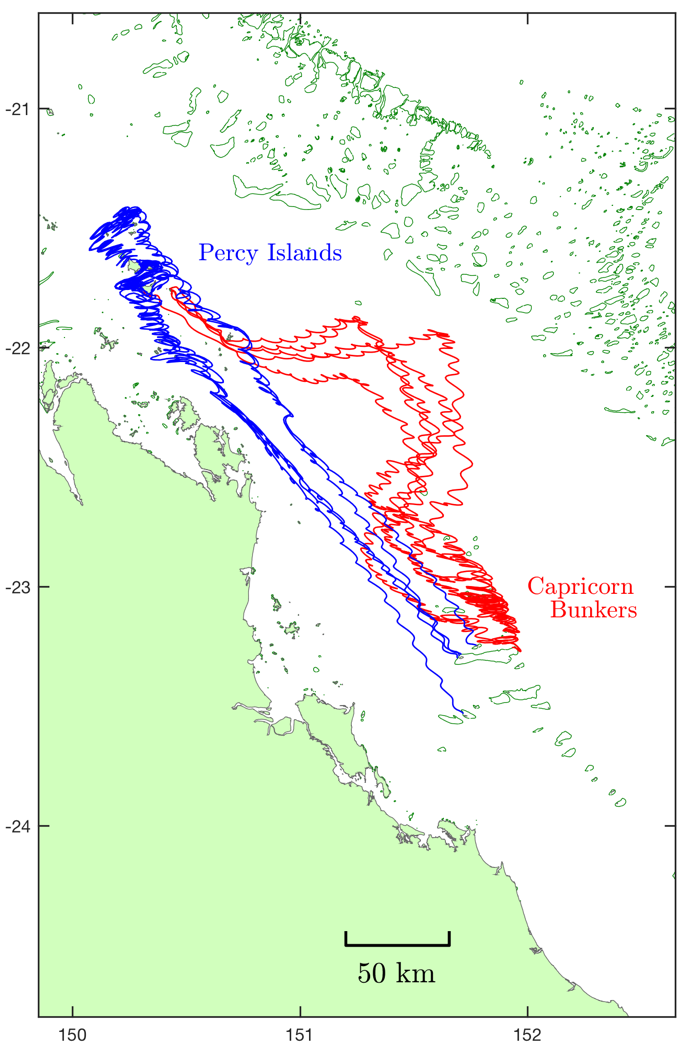


*Fig S3.3: Multiple dispersal trajectories linking the Capricorn Bunker group to the Percy Island group. These trajectories were generated by the consistent larval behavioural model, during a single spawning event centred around 26/07/2011, when bidirectional exchange was observed in the parentage assignment dataset. Red larvae were spawned at the Capricorn Bunker group, while blue larvae were spawned on the Percy Island group. Larvae in the genetic parentage dataset were also observed to connect these two reefs. Note that larvae from the Capricorn Bunker group are using offshore northward-flowing currents, while larvae from the Percy Island group are using inshore southward-flowing currents (after an initial period where they stayed in the vicinity of their natal reefs).*

The process of dispersal event matching also provides an explanation for some of the unusual events observed in the genetic dataset. For example, parentage assignments indicate that dispersal occurred in both directions between the Percy Island group and the Capricorn Bunker group in the spawning event that occurred on the 26/07/2011 full moon. Thus, reefs that are more than 200 km apart were connected by dispersal in both directions. The biophysical simulations also include these bidirectional dispersal events, and closer inspection reveals how. The larvae travelling north are being transported by offshore northward currents, while the larvae travelling south are being transported by inshore southward currents (Fig S3.3). This same larval dispersal event is also shown in an animation (*S1 Animation* ).

**Likelihood fitting**

Here, we develop a function that calculates the likelihood that a genetic parentage assignment dataset was generated by a particular biophysical model, given both the population processes and the empirical sampling processes that led to the parentage dataset.

We begin with a series of definitions. For a given spawning event $t$, we assume that $p_{ijt}$describes the probability that a larvae spawned on reef $i$ will disperse, survive, and settle on reef $j$. Note that, because most larvae do not survive the dispersal process, $\sum_{j} p_{ijt}<1$ for all *i*. Across all *M* reefs in a system, these probabilities combine to create a true connectivity matrix for each spawning event:

$$\mathbf{C}_{t}=\left[ \begin{matrix} p_{11t} & \ldots& p_{1Mt} \\ \vdots& \ddots& \vdots\\ p_{M1t} & \ldots& p_{MMt} \end{matrix} \right] .$$

Equation S3.1

Our goal was to construct a biophysical larval dispersal simulation model that creates connectivity matrices that match the true connectivity matrices. Biophysical models do not directly estimate probabilities. Instead, they simulate a large number of individual larval dispersal events. We assume that with enough releases, the simulations create an approximate connectivity matrix:

$$\mathbf{C}_{t}^{k}=\left[ \begin{matrix} \frac{m_{11t}}{r_{1}} & \ldots& \frac{m_{1Mt}}{r_{1}} \\ \vdots& \ddots& \vdots\\ \frac{m_{M1t}}{r_{M}} & \ldots& \frac{m_{MMt}}{r_{M}} \end{matrix} \right] .$$

Equation S3.2

where $k$ identifies the biophysical model we are assessing: either $\mathbf{C}_{t}^{F}$ for the consistent model, $\mathbf{C}_{t}^{E}$ for the varying model, and $\mathbf{C}_{t}^{P}$ for the passive model. In Eq. S3.2, $m_{ijt}$ is the number of simulated larvae from spawning event *t* that travel from reef *i* to reef *j*, and $r_{i}$ is the total number of larvae released from reef *i* each simulated dispersal event*.* Note that, as with the true connectivity matrix, $\sum_{j} m_{ijt}\leq r_{i}$, because many larvae die during their simulated dispersal, and others never encounter suitable settlement habitat. Essentially, we’re claiming that $\frac{m_{ijt}}{ri}\approx p_{ijt}$.

Our goal is to choose a biophysical model that creates simulated connectivity matrices $\mathbf{C}_{t}^{k}$ which match the true connectivity matrices $\mathbf{C}_{t}$. Unfortunately, we don’t have access to the true connectivity matrix. Instead, we have an observed dataset of genetic parentage assignments:

$$\mathbf{B}_{t}=\left[ \begin{aligned} \begin{matrix} b_{11t} & \ldots& b_{1Mt} \end{matrix} \\ \begin{matrix} \vdots& \ddots& \vdots\end{matrix} \\ \begin{matrix} b_{M1t} & \ldots& b_{MMt} \end{matrix} \\ \begin{matrix} b_{x1t} & \ldots& b_{xMt} \end{matrix} \end{aligned} \right] ,$$

Equation S3.3

where $b_{ijt}$ is the number of juveniles sampled on reef *j* at time *t* that were assigned to sampled parents on reef *i*, and $b_{xjt}$ is the number of juveniles sampled on reef *j* that could not be assigned to any sampled adults in the dataset.

The parentage dataset looks similar to a connectivity matrix, but direct comparisons between $\mathbf{B}_{t}$ and $\mathbf{C}_{t}^{k}$are not possible because $\mathbf{B}_{t}$is separated from the connectivity matrix by a complicated, three-step sampling process. First, the parentage dataset is based on a finite sample of juveniles from the population on each sampled reef. Second, the parentage dataset can only assign those juveniles if one of their parents was a sampled adult, and most cannot be assigned. Third, many settling larvae will not survive to become recruits (and therefore to be sampled), and so the recruitment cohort is itself a sample of the settling cohort. These sampling processes mean that, even when many juveniles are assigned to a particular source reef, it doesn’t follow that the probability of a larvae dispersing between those patches $p_{ijt}$ is large (Eq. S3.1). Instead, a large number of assignments from juveniles on reef *j* to adults on reef *i* could indicate that:

1. The population of adults on reef *i* is particularly large, and produces a large number of larvae.
2. The proportion of adults that were sampled on reef *i* was high, meaning that more of the dispersal events between the two reefs could be successfully assigned.
3. The number of juveniles sampled on reef *j* is particularly large, and more connections of all types will be observed.
4. The number of larvae that disperse from reef *i* to reef *j* is small, but still larger than all other larval connections to reef *j*. This makes a sample on reef *j* likely to be from a parent on reef *i*.
5. The probability $p_{ijt}$ is particularly large*.*

Our likelihood function accounts for each of these possibilities.

***Likelihood function***

The likelihood of observing the parentage dataset $\mathbf{B}_{t}$ given a particular biophysical model (denoted $\theta$) is:

$$LL\left( \theta\mathbf{|}\mathbf{B}_{t} \right)=\sum_{j=1}^{M} b_{xjt}\ln\left( \frac{\left( 1-\pi_{i} \right)^{2}N_{i} \frac{m_{ijt}}{r_{i}}}{\sum_{a=1}^{P} N_{a}\frac{m_{ajt}}{r_{a}}} \right)+\sum_{i,j=1}^{M} b_{ijt}\ln\left( \frac{\left( 1-\left( 1-\pi_{i} \right)^{2} \right)N_{i} \frac{m_{ijt}}{r_{i}}}{\sum_{a=1}^{P} N_{a}\frac{m_{ajt}}{r_{a}}} \right).$$

Equation S3.4

Note that multinomial likelihood functions normally include multinomial coefficients, but these will be the same for all models and therefore do not affect the relative fit. Moreover, their large size can cause quantisation (i.e., rounding) errors, and so omitting them is generally a good idea, and we omitted them from our likelihood function. In Eq. S3.4, $\pi_{i}$ is the proportion of adults sampled on each reef *i* from a total population of $N_{i}$. The term in the first set of parentheses is therefore the probability that a sampled juvenile on patch *j* is the offspring of two unsampled parents (as recorded in $b_{xjt}$). The term in the second set of parentheses is the probability that a sampled juvenile on patch *j* is the offspring of at least one sampled parent on patch *i* (as recorded in $b_{ijt}$).

Both terms in the likelihood function depend critically on the vector of adult populations $N_{i}$, since large adult populations amplify the effects of the modelled dispersal probabilities $m_{ijt}/r_{i}$. During the adult sampling process, adult populations were estimated on each of the sampled reefs, along with their normally-distributed mean and standard error $N_{i}\sim\mathrm{Normal}\left( \bar{N}_{i},s_{i} \right)$. Sampled populations included both protected and unprotected reefs – in the GBR, the former are Marine National Park reefs, which are no-take reserves; the latter are either Conservation Park Zones (CPZ) or Habitat Protection Zones (HPZ) , where fishing is allowed under gear and catch restrictions. For the purposes of this analysis, we treated CPZ and HPZ as equivalent, since fishing for *P. maculatus* is allowed in both. Fishing restrictions for *Plectropomus* species are lighter in CPZ (1 hook per person) than in HPZ (maximum 6 hooks per person), but bag limits (< 7 per angler) and size limits (> 38 cm) apply in both. For unsampled reefs, we assumed that adult abundance was proportional to reef area, at a density equal to the average sampled reef in that region (e.g., the Capricorn Bunker group), with similar protection status. We propagated this uncertainty about adult abundance by repeatedly estimating the maximum likelihood kernel parameters, using adult population sizes that were randomly sampled from the distributions on each reef.

*P. maculatus* is an inshore specialist, with sizeable populations on midshelf reefs but almost no individuals on offshore reefs, where its congenerics *P. leopardus* and *P. laevis* dominate. We took this distribution into account when considering the potential larval contributions from offshore reefs in the southern GBR (specifically the Swains and Strong Tidal mid-shelf reefs, which sit on the far side of the Capricorn Channel from the study reefs). Specifically, we considered the populations on the inner shelf, mid-shelf and outer shelf reefs to be 100%, 50% and 5% *P. maculatus*, in line with observations*.* Since none of these populations were sampled, they only contribute to the unsampled juvenile proportions, and did not have a strong influence on the model fits.

The genetic parentage data was sampled in two consecutive spawning seasons (2011 and 2012), with each sample representing approximately a year of settlement (*S1 Text*; Fig S1.1). We therefore split the observed parentage data into two separate dispersal and sampling events ($\mathbf{B}_{1}$ and $\mathbf{B}_{2}$), and likewise created two biophysical connectivity matrices $\mathbf{C}_{1}^{k}$ and $\mathbf{C}_{2}^{k}$, each the aggregate of simulated dispersal events across a 12 month period. Because *P. maculatus* spawning density varied consistently throughout the year, we constructed the biophysical connectivity matrices by weighting each month according to the observed spawning density function $S(t)$:

$$m_{ij, 2011}=S\left( Jan 11 \right)\cdot m_{ij,Jan 2011}+S\left( Feb 11 \right)\cdot m_{ij,Feb 2011}+\ldots+S\left( Jul 13 \right)\cdot m_{ij, Jul 2011},$$

Equation S3.5

and similarly for $m_{ij,2012}$. The overall log likelihood of each biophysical model was the sum of the log likelihood of the two years of sampling:

$LL\left( \mathbf{C}_{t}^{k}|\mathbf{B}_{t} \right)=LL\left( \mathbf{C}_{1}^{k}|\mathbf{B}_{\mathbf{1}} \right)+LL\left( \mathbf{C}_{2}^{k}|\mathbf{B}_{\mathbf{2}} \right)$.

Equation S3.6

Using these methods, we calculated the likelihood of observing the southern GBR parentage dataset using three different biophysical models: the consistent model $LL\left( \mathbf{C}_{t}^{F}|\mathbf{B}_{t} \right)$, the varying model $LL\left( \mathbf{C}_{t}^{E}|\mathbf{B}_{t} \right)$, and the passive model $LL\left( \mathbf{C}_{t}^{P}|\mathbf{B}_{t} \right)$. Of the three models, the consistent biophysical model consistently had the highest likelihood, given the observed parentage data, followed by the passive model, and then the varying model, $LL\left( \mathbf{C}_{t}^{F}|\mathbf{B}_{t} \right)> LL\left( \mathbf{C}_{t}^{P}|\mathbf{B}_{t} \right)> LL\left( \mathbf{C}_{t}^{E}|\mathbf{B}_{t} \right)$, as shown in main text Fig 2A.

***Goodness-of-fit test***

The fact that the consistent model provides the maximum likelihood fit to the data does not mean that it offers a “good” fit to the data. To assess the goodness-of-fit of the consistent biophysical model, we undertook a parametric bootstrap goodness-of-fit (PB-GOF) test.

The theory behind the PB-GOF test is as follows. The log likelihood of the consistent biophysical model is approximately $LL\left( \mathbf{C}_{t}^{F}\boldsymbol{|}\mathbf{B}_{t} \right)=-518.2$ (the blue histogram in main text Fig 2A) – the best model in our set. We want to ask: does a value of $-518.2$ suggest that the consistent model is providing an accurate representation of the true connectivity in the system? To assess this, we calculate what the value of the log likelihood statistic would be if the consistent model were the true model. To estimate this test statistic distribution, we repeatedly simulate versions of our observed dataset $\mathbf{B}_{1}^{F}$ and $\mathbf{B}_{2}^{F}$, by sampling from the consistent biophysical model connectivity matrices $\mathbf{C}_{t}^{F}$, that is, we generate $\mathbf{B}_{1}^{F}\boldsymbol{(}\mathbf{C}_{1}^{F}\boldsymbol{)}$, and $\mathbf{B}_{2}^{F}\boldsymbol{(}\mathbf{C}_{2}^{F}\boldsymbol{)}$. We then calculate the likelihood of each of these datasets, given the consistent biophysical model: $LL\left( \mathbf{C}_{t}^{F}\boldsymbol{|}\mathbf{B}_{t}^{F}\left( \mathbf{C}_{t}^{F} \right) \right)$. Note the recursive nature of this likelihood: “what is the likelihood of the consistent biophysical model, given a dataset generated by the consistent biophysical model?”. We are trying to estimate what the likelihood would look like if we were fitting a parentage dataset with the true model.

By repeatedly generating and fitting sample matrices $\mathbf{B}_{t}^{F}$**,** we are able to construct a distribution of expected log likelihoods; these are shown in grey in Fig 2A of the main text. The distribution has a lower 95% confidence bound of $-605$, indicating that the log likelihood of the consistent biophysical model ($-518.2$) represents a good fit to the genetic parentage data.

***Power analysis***

To assess the expected classification performance of (1) the likelihood equation and (2) our parentage assignment dataset, we ran a series of simulated fitting exercises using the three biophysical models.

For each of 1,500 replicates, we simulated a sample parentage dataset from one of the three biophysical models (consistent, varying, and passive) of the same size as the observed dataset (i.e., with the same proportion of adults sampled on each reef, the same number of juveniles sampled at each reef, and 69 positive assignments). The simulated samples were taken from the same set of southern GBR reefs as the observed parentage data.

We then used the likelihood equation to guess which of the three biophysical simulations models generated the data (i.e., had the maximum likelihood). The result was the confusion matrix shown in Table S3.1, which indicates that the likelihood equation and parentage sampling scheme is able to identify the correct underlying model in 83% of cases. Discrimination was highest when the consistent and passive biophysical models were used to generate the data, and lowest when the varying model was used. In this case, the varying model was mistaken for the consistent model in 42% of simulations.

*Table S3.1: Confusion matrix for the power analysis of the likelihood equation and parentage assignment dataset. The rows indicate the model used to generate the sample dataset, the columns indicate the model associated with the maximum likelihood, and the value in each cell indicates the proportion of 500 samples that returned a particular combination. The model was best at discriminating the passive and consistent models, but it frequently mistook the varying model for the consistent model.*

|  | | Maximum likelihood model | | |
| --- | --- | --- | --- | --- |
|  |  | Passive | Varying | Consistent |
| True model | Passive | 0.94 | 0.00 | 0.06 |
|  | Varying | 0.00 | 0.58 | 0.42 |
|  | Consistent | 0.01 | 0.02 | 0.97 |

**Inter- versus intra-regional biophysical model matching**

In Fig 2B in the main text, we produce a scatter plot of observed dispersal versus predicted dispersal for the consistent biophysical model (the maximum likelihood model). The scatter suggests that the consistent model has a tendency to overestimate intra-regional dispersal and underestimate inter-regional exchange. Here, we decompose the scatterplot further, and compare the performance for the three different biophysical models we compared with the parentage datasets, while cautioning that visual inspection is not adequate to assess model fit to a complex dataset in the presence of sampling uncertainty.

In Fig S3.4 we show the inter- and intra-regional exchanges in different subplots, for all three models. It is clear that the varying model does a very poor job at predicting inter-regional exchanges, predicting almost no successful dispersal between regions where such events were observed. The passive model does a better job at predicting inter-regional exchanges, but still underestimates a lot of observed connections. It also underestimates most intra-regional dispersal events.


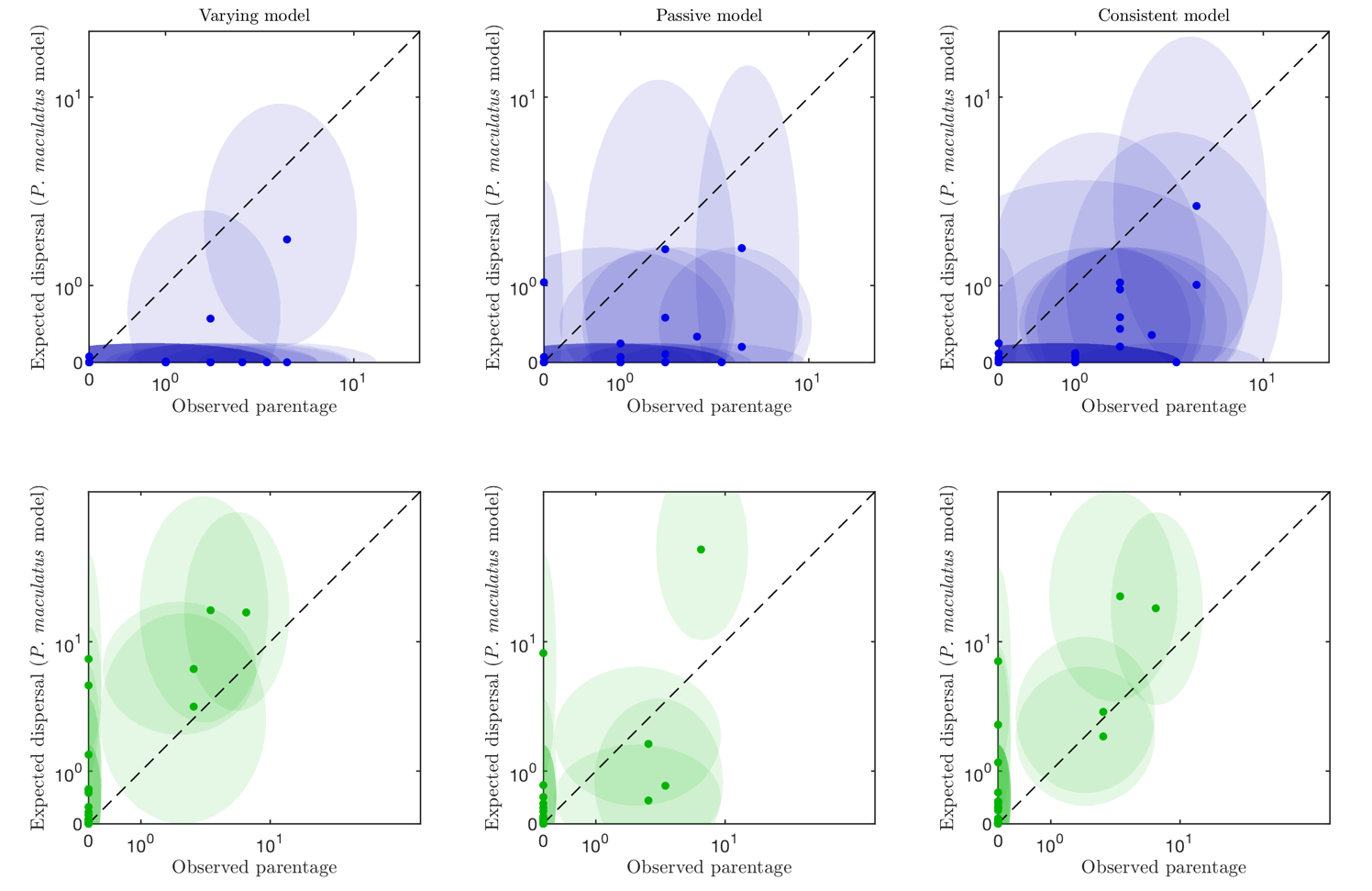


*Fig S3.4. Scatter plots of expected and observed connections between pairs of reefs that are in the same region (green markers) and are in different regions (blue markers). To ensure that overlapping markers are visible, we have used transparency and have added small amounts of normally distributed variation to the x- and y-position of each marker. The dashed lines indicate an accurate 1:1 prediction.*

None of the models predict the observed dispersal perfectly, but it is important to remember that the markers in Fig 2B and Fig S3.4 show observations of multinomial sampling events, often with a small number of events. This is the source of the uncertainty halos shown around each marker. We expect these halos to be relatively large – if we tossed a fair coin 4 times, we would not necessarily expect the observed proportion of heads to perfectly match the expected proportion (2 out of 4). We intuitively accept that stochastic variation makes 3 heads relatively likely (with a 25% chance), and even allows for zero heads (with a 6.25% chance). The same tolerance of variation from expected outcomes should be applied to parentage assignments, particularly for small-sample outcomes near the origin of the scatterplots.

**Directional matching**


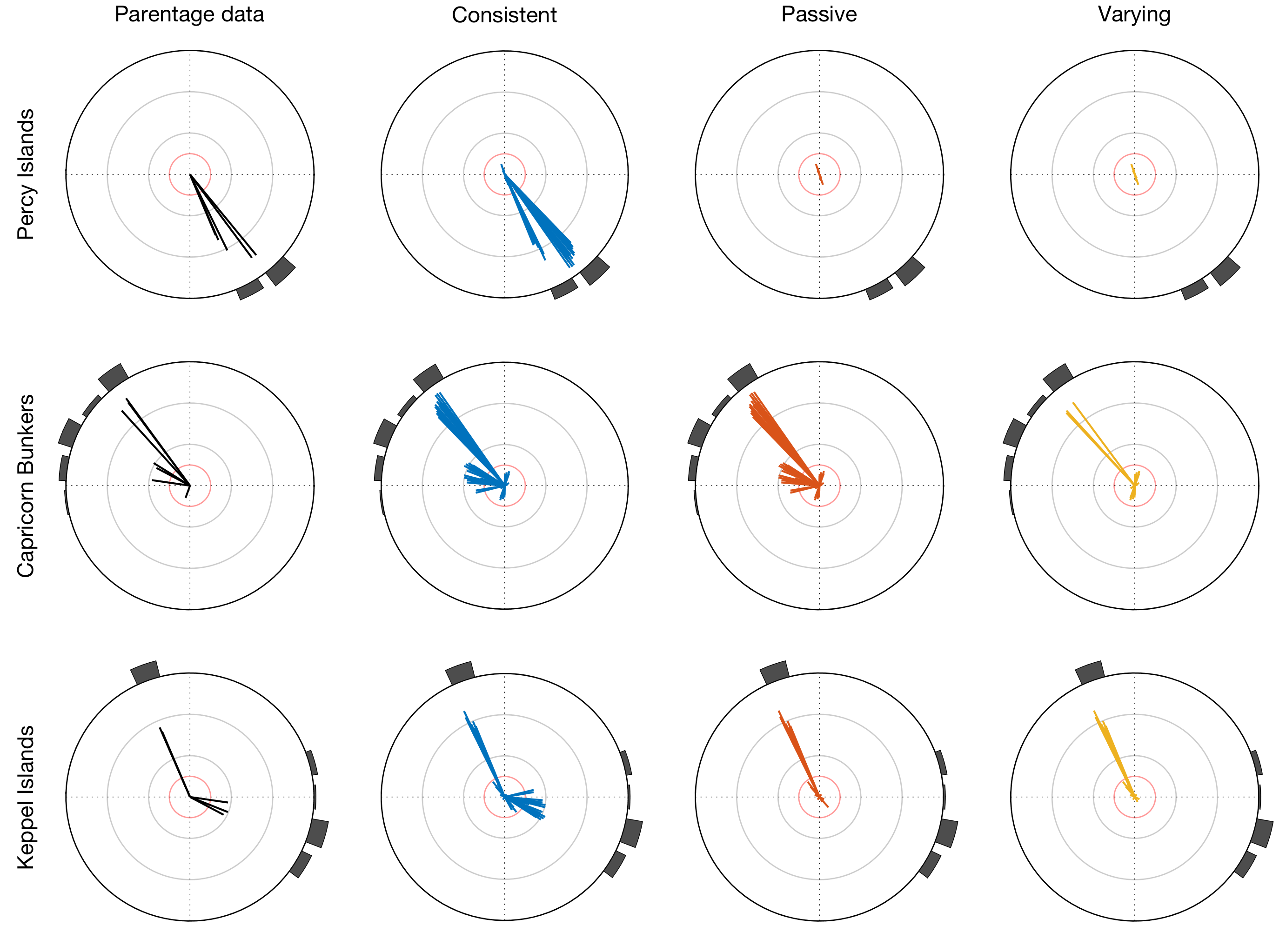


*Fig S3.5. Compass plots illustrating the direction and distance travelled by all larvae in the genetic parentage assignment data, and in the three biophysical models. In each panel the concentric circles measure distances of 50 km (red circle), then 100 km, 200 km, and 300 km (outside circle). The histograms around the outside indicate the relative amount of sampled habitat in the two other reef groups (these constrain where larvae could have settled). Note that the straight lines are not meant to suggest that the dispersal trajectories themselves are straight lines, as shown in Fig S3.1 & Fig S3.3.*

We also sought to compare the ability of the different models to recreate the patterns of directional dispersal observed in the parentage assignment data. Each of the three sampled regions (the Keppel Island group, the Percy Island group, and the Capricorn Bunker group) had quite different directional patterns of dispersal. This is unsurprising, because the oceanographic context varies considerably between these regions, as does the surrounding habitat distribution (which constrains the possibility of observing any dispersal).

We therefore produced separate compass plots of dispersal for each reef group, describing the distance and direction of dispersal from each of the reefs in that group. For each parentage assignment in the observed dataset, and for each simulated biophysical trajectory (between two sampled reefs), we draw a polar vector beginning at the origin, whose radial length describes the distance between the source and destination reefs, and whose angle reflected the compass direction from source to destination. The results for the parentage data and the consistent model are shown in the main text Fig 3B, and for all datasets in Fig S3.5.

The compass plots in Fig S3.5 show that the consistent biophysical model can recreate the directional dispersal patterns in each of the three sampled reef regions. The other two models – the varying and passive models – can recreate some elements of the observed patterns, but not all. Specifically, both the passive and varying models reproduce the dispersal observed from source reefs in the Capricorn Bunker group, suggesting that this dispersal (particularly the north-westerly dispersal to the Percy Island group) is the result of oceanographic forcing, rather than larval behaviour. In contrast, neither model can reproduce the southward dispersal from the Percy Island group, nor the south-easterly dispersal from the Keppel Island group to the Capricorn Bunker group. These observed dispersal trajectories require particular larval behavioural decisions.

**Dispersal kernel matching**

Larval dispersal kernels express the amount of larvae that disperse a given distance *d* as an isotropic function $p\left( d \right)$. Bode et al. [1] published methods that fit kernels to genetic parentage data, while accounting for the heterogeneous distribution of habitat, the sampling distribution of adults, and the presence of unsampled adults both on the sampled reefs and on entirely unsampled reefs (“ghost populations”). We apply these methods in these analyses, with one variation.

The likelihood function for fitting kernels to the parentage data estimates the proportional contribution of reef $i$ to the settlement cohort on reef $j$ to be:

$$q_{ij}\left( \boldsymbol{\theta} \right)=\frac{\left( 1-\left( 1-\pi_{i} \right)^{2} \right)N_{i} \rho\left( d_{ij},\boldsymbol{\theta} \right)}{\sum_{a=1}^{P} N_{a} \rho\left( d_{aj},\boldsymbol{\theta} \right)}.$$

Equation S3.7

These probabilities depend on the size of the adult populations ($N_{i}$). Our empirical data estimates both the average size of the adult population on each reef $i$ ($\bar{N}_{i}$), and also the standard error in this estimate ($s_{i}$). In the procedure outlined in Bode et al. (2018), we used only the mean adult population when estimating larval output from each population. When constructing confidence bounds around our best-estimate dispersal kernels in these analyses, we repeatedly estimate the maximum likelihood kernel parameters for adult population sizes that were randomly sampled from the distributions: $N_{i} \sim\mathrm{Normal}\left( \bar{N}_{i}, s_{i} \right)$.

It is challenging to graphically represent the uncertainty associated with larval dispersal kernels. When expressed as probability density functions, dispersal kernels are normalised such that their integral is one:

$$\hat{p}\left( d \right)=\frac{p\left( d \right)}{2\pi\int_{x=0}^{\infty} p\left( x \right)\cdot dx}.$$

Equation S3.8

If this condition is also applied to the upper and lower confidence bounds, as generated by the 95% intervals of the model parameters, the result is a distance at which uncertainty is shown to be zero, and a section where the mean kernel sits outside the confidence bounds. These are clearly poor representations of the uncertainty. See Fig S3.6A, and Fig 2 of d’Aloia et al. [2] for examples of this.

An alternative is to express the amount of dispersal as relative to local retention:

$$\hat{p}\left( d \right)=\frac{p\left( d \right)}{p\left( 0 \right)}.$$

Equation S3.9

While this description of uncertainty has the benefit of capturing how the decay rates of dispersal vary between the confidence bounds, it creates a different zero-uncertainty location at $d=0$, where local retention occurs (Fig S3.6B). This is undesirable, as there is no reason that this part of the domain will have lower uncertainty. Moreover, it means that the kernels are no longer probability distributions, which makes comparisons between alternative kernels more difficult. For example, kernels that predict a smaller proportion of short-distance dispersal should predict a larger proportion of long distance dispersal. This trade-off is not visible if we use Equation S3.9. See Almany et al. [3] for an example of this representation of uncertainty.

Both depictions of the kernel bounds therefore capture different elements of uncertainty, but are dissatisfying in unique ways. We therefore choose to include both descriptions in our confidence bounds, by selecting the widest of the four confidence bounds at all distances from the natal reef (Fig S3.6C). This approach has the benefit of leaving the mean dispersal kernel (denoted by the solid line) as a probability density function, while allowing the confidence bounds to visually represent the degree of uncertainty that exists at both short and long distances from the natal reef. We use this description in Fig 3 in the main text.


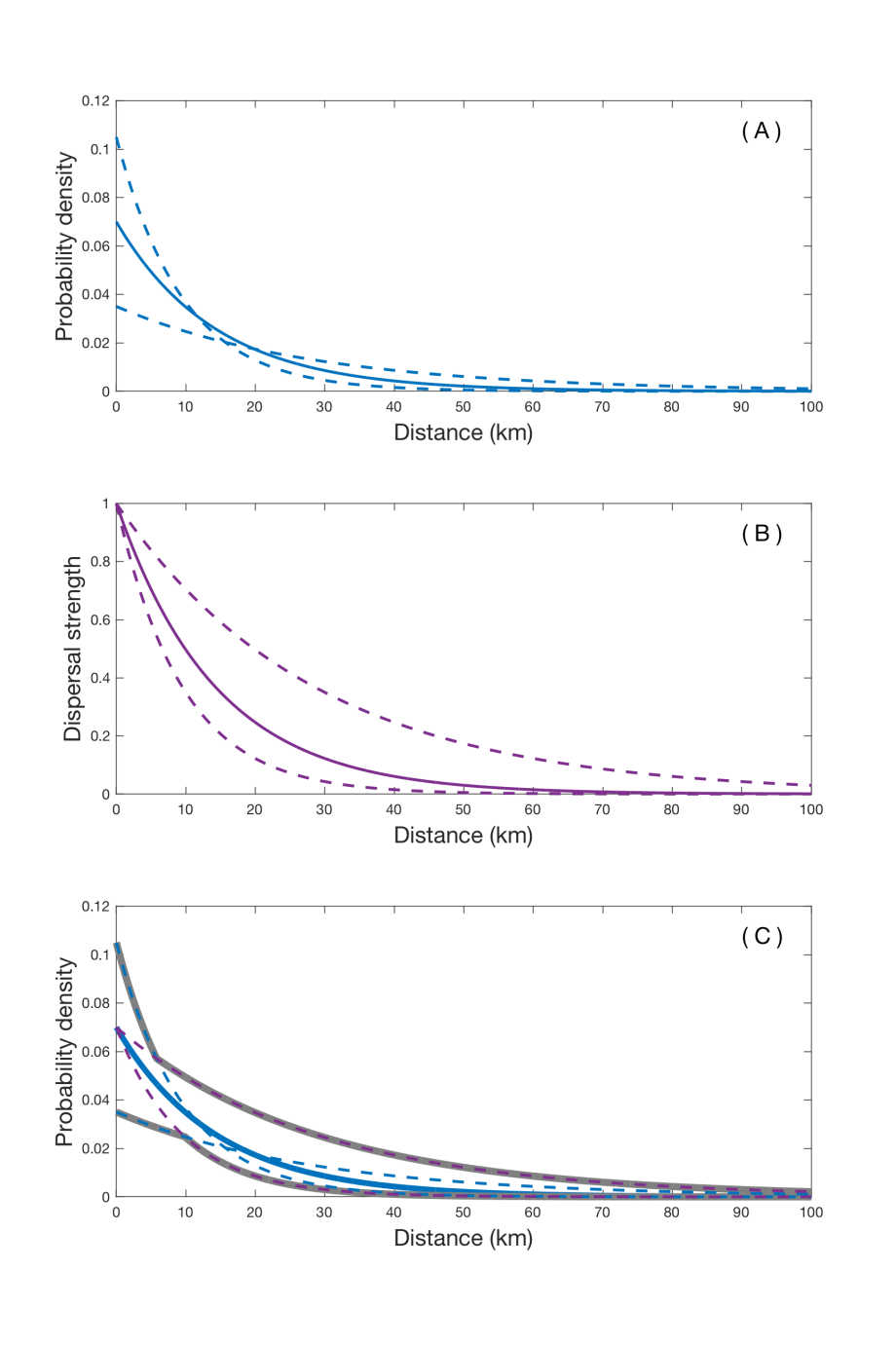


*Fig S3.6: Three descriptions of uncertainty in larval dispersal kernels for the Laplacian kernels*

$$p\left( d \right)=A\exp\left[ -kd \right].$$

**(A)** *The parameter* $A=k$*is chosen to make* $p\left( d \right)$ *a probability density function. Solid line shows using the mean value of* $k$*; dashed lines show the 95% confidence bounds of the parameter values. Note that the upper and lower confidence bounds intersect at* $d=15$ *km, and that at this point they do not enclose the mean value.* **(B)** *The parameter* $A=1$ *is chosen to show dispersal strength of all three lines relative to their local retention* $p\left( 0 \right)$*. Note that the confidence bounds intersect at* $d=0$*, communicating a false certainty about the relative strength of local retention.* **(C)** *A representation of the dispersal kernel where the average value is a probability distribution* $p\left( d \right)=\bar{k}\exp\left[ -\bar{k}d \right]$*, as in panel (A). The confidence bounds in this case are the maximum and minimum values observed across panels (A) and (B).*

**Matrix correlations**

Matrix correlations have previously been used to compare biophysical models with connectivity matrices measured using genetic and chemical assignment tests. Both matrices are expressed as ordered vectors (i.e., the matrix is “unrolled” into a single column vector), and the linear relationship between the two is measured using Pearson’s correlation coefficient. For our simulations, we found a consistently high correlation between all three biophysical models and the parentage data: Pearson’s *r* = 0.97 (95% CIs = [0.94, 0.98]) for the consistent model, *r* = 0.96 [0.94, 0.98] for the varying model, and *r* = 0.97 [0.95, 0.99] for the passive model. These confidence intervals were constructed by creating multiple sample matrices of equivalent size to the observed parentage assignment matrix.

While these high correlation statistics indicate that the two sets of matrices look alike, they primarily indicate the low statistical power offered by matrix correlation as a tool or assessing goodness of fit for biophysical models to genetic parentage or assignment data. Given the substantially different predictions made by the various models in other ways (see above), it’s clear that this goodness-of-fit test has difficulty discriminating between good and poor models.

**References**

1. Bode, M., Bode, L., Choukroun, S., James, M. K., & Mason, L. B. (2018). Resilient reefs may exist, but can larval dispersal models find them?. *PLoS Biology*, *16*(8), e2005964.
2. D’Aloia, C. C., Bogdanowicz, S. M., Francis, R. K., Majoris, J. E., Harrison, R. G., & Buston, P. M. (2015). Patterns, causes, and consequences of marine larval dispersal. *Proceedings of the National Academy of Sciences*, *112*(45), 13940-13945.
3. Almany, G. R., Hamilton, R. J., Bode, M., Matawai, M., Potuku, T., Saenz-Agudelo, P., ... & Russ, G. R. (2013). Dispersal of grouper larvae drives local resource sharing in a coral reef fishery. *Current biology*, *23*(7), 626-630.
