# Supplementary figures and images for: Successful validation of a larval dispersal model using genetic parentage data
Source: PLoS Biol. 2019 Jul 12;17(7):e3000380. doi: 10.1371/journal.pbio.3000380 (PMC6655847; doi:10.1371/journal.pbio.3000380)

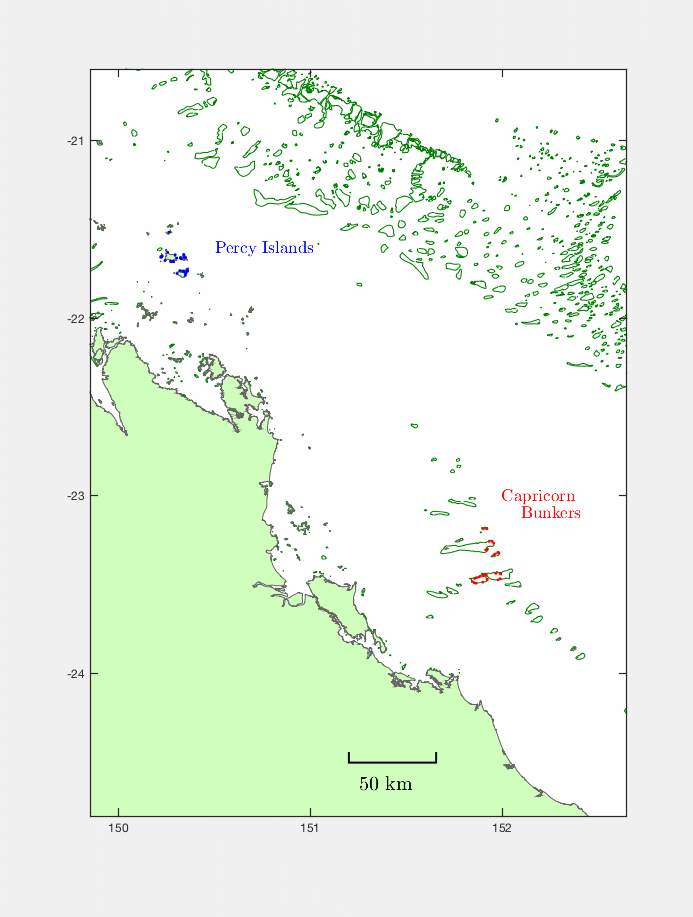

Supplement: S1 Animation — Simulations are generated by the consistent biophysical model, recreating the 26/07/2011 spawning event. Red larvae were spawned in the Capricorn Bunker group; blue larvae were spawned in the Percy Islands. Note that these larvae were selected for illustrative purposes and are neither all the larvae that were released from these two reef groups nor the only ones that travelled between them. (GIF) [file pbio.3000380.s004.gif]
